# Whole-Transcriptome Analysis Unveils the Synchronized Activities of Genes for Fructans in Developing Tubers of the Jerusalem Artichoke

**DOI:** 10.3389/fpls.2020.00101

**Published:** 2020-02-21

**Authors:** Marco Bizzarri, Massimo Delledonne, Alberto Ferrarini, Paola Tononi, Elisa Zago, Doriano Vittori, Francesco Damiani, Francesco Paolocci

**Affiliations:** ^1^Department of Science and Technology for Agriculture, Forests, Nature and Energy (DAFNE), University of Tuscia, Viterbo, Italy; ^2^Department of Biotechnology, University of Verona, Verona, Italy; ^3^Institute of Bioscience and Bioresources (IBBR), National Research Council (CNR), Perugia, Italy

**Keywords:** *Helianthus tuberosus*, tuber phenology, inulin, fructosyltransferases, expressed sequence tags, microarray, qRT-PCR

## Abstract

*Helianthus tuberosus* L., known as the Jerusalem artichoke, is a hexaploid plant species, adapted to low-nutrient soils, that accumulates high levels of inulin in its tubers. Inulin is a fructose-based polysaccharide used either as dietary fiber or for the production of bioethanol. Key enzymes involved in inulin biosynthesis are well known. However, the gene networks underpinning tuber development and inulin accumulation in *H. tuberous* remain elusive. To fill this gap, we selected 6,365 expressed sequence tags (ESTs) from an *H. tuberosus* library to set up a microarray platform and record their expression across three tuber developmental stages, when rhizomes start enlarging (T_0_), at maximum tuber elongation rate (T_3_), and at tuber physiological maturity (T_m_), in “VR” and “K8-HS142”clones. The former was selected as an early tuberizing and the latter as a late-tuberizing clone. We quantified inulin and starch levels, and qRT-PCR confirmed the expression of critical genes accounting for inulin biosynthesis. The microarray analysis revealed that the differences in morphological and physiological traits between tubers of the two clones are genetically determined since T_0_ and that is relatively low the number of differentially expressed ESTs across the stages shared between the clones (93). The expression of ESTs for *sucrose:sucrose 1-fructosyltransferase* (*1-SST*) and *fructan:fructan 1-fructosyltransferase* (*1-FFT*), the two critical genes for fructans polymerization, resulted to be temporarily synchronized and mirror the progress of inulin accumulation and stretching. The expression of ESTs for starch biosynthesis was insignificant throughout the developmental stages of the clones in line with the negligible level of starch into their mature tubers, where inulin was the dominant polysaccharide. Overall, our study disclosed candidate genes underpinning the development and storage of carbohydrates in the tubers of two *H. tuberosus* clones. A model according to which the steady-state levels of *1-SST* and *1-FFT* transcripts are developmentally controlled and might represent a limiting factor for inulin accumulation has been provided. Our finding may have significant repercussions for breeding clones with improved levels of inulin for food and chemical industry.

## Introduction

Although starch is the most common reserve carbohydrate in higher plants, about 15% of all flowering plant species store fructans. These are linear and branched polymeric fructose extensions of sucrose (Suc) (Suzuki and Chatterton, 1993). Widespread in cereals (i.e., barley, wheat, and oat) (Pollock and Cairns, 1991), vegetables (i.e., chicory, onion, and lettuce), ornamentals (i.e., *Dahlia* spp. and tulip), and forage grasses (i.e., *Lolium* and *Festuca*) (Hendry, 1993), fructans are located in the vacuole and are water-soluble. Conversely, starch is insoluble and stored in the amyloplasts, or temporarily, in the chloroplast.

The storage capacity of vacuoles may be larger than that of the plastids, accounting for up to 95% of the protoplast volume. Therefore, the storage capacity of fructans is higher than that of starch (Brocklebank and Hendry, 1989), primarily when associated with the formation of specialized organs such as succulent stems, bulbs, and tubers.

Inulin is a fructan that displays mostly or exclusively the linear 2→1 fructosyl-fructose linkage between the *β*-D-fructosyl units (Glu _1←2_ Fru _1←2_ Fru*_n_*, where *n* ranges from 1 to 33) (Suzuki and Chatterton, 1993). Usually, it is formed in plant organs of species belonging to the order Asterales and within grasses. Asterales include species that produce inulin-rich biomass either in roots as it occurs in *Cichorium intybus* (Li et al., 1997) or in auxotrophic stems and tubers as in *Helianthus tuberosus* L. (Kays and Nottingham, 2007; De Pace et al., 2010). The former is by far the most commonly used source of inulin by the food industry for its interesting nutritional, health-promoting, and technological properties (Flamm et al., 2001; Schaafsma and Slavin, 2015). However, inulin as much as other fructans can be converted into bioethanol through microbial fermentation (Martel et al., 2010).

Fructan-accumulating species that produce biomass with low input of fertilizers, pesticides, and carbon footprint are thus ecologically sustainable candidates to replace staple and starch-rich crops for bioethanol production.

*H. tuberosus*, also known as the Jerusalem artichoke (2n=6x=102), is a perennial rhizomatous species, adapted to low-nutrient soils. It exhibits high nitrogen and water-use efficiency (Mecella et al., 1996; Zhao et al., 2005; Kays and Nottingham, 2007), good competitive ability against weeds (Wall et al., 1987; Paolini and De Pace, 1997; Kays and Nottingham, 2007), and tolerance to diseases (Cassels and Walsh, 1995). These features coupled to the fact that the content of inulin in the tubers can be up to 30% on a fresh weight make *H. tuberosus* an ideal feedstock for ethanol production under different cropping systems, particularly in marginal lands (Kays and Nottingham, 2007).

Critical enzymes involved in inulin biosynthesis in *H. tuberosus* as well as other species are well known. The model of fructan biosynthesis has been reviewed and perfected since 1968 (Edelman and Jefford, 1968; Suzuki and Chatterton, 1993; Bonnett et al., 1994; Vijn and Smeekens, 1999; Altenbach and Ritsema, 2007). Biosynthesis of plant fructans requires specific enzymes like fructosyltransferases, which catalyze in the vacuole the transfer of fructosyl units from a donor substrate (sucrose or fructan oligosaccharides) to an acceptor substrate (sucrose or fructan oligosaccharides). Synthesis is always initiated by the sucrose:sucrose 1-fructosyltransferase (1-SST) from two molecules of sucrose, producing the shortest glucose (Glu)-Fru fructan chain “Glu _1←2_ Fru _1←2_ Fru” (or GF_2_), called 1-kestose. In this case, sucrose serves as both a fructosyl donor and acceptor. The second step involves the fructan:fructan 1-fructosyltransferase (1-FFT) which drives the fructan chain elongation by adding a fructose residue from 1-kestose (GF_2_) or 1,1-nystose (GF_3_) or a fructan molecule with a degree of polymerization (DP) higher than 3 (GF > 3) to other fructan molecules with DP > 3 (Edelman and Jefford, 1968; Koops and Jonker, 1994; Koops and Jonker, 1996).

The aims of *H. tuberosus* breeders are primarily to enhance the yield in tubers and the inulin content therein. However, despite the wealth of knowledge on the enzymology of inulin in *H. tuberosus*, the gene networks underpinning tuber development and inulin accumulation therein have yet to be fully elucidated. There are only few studies aimed at unearthing the transcriptional profiles of genes involved in carbohydrate accumulation and metabolism in these organs (Jung et al., 2014; Jiao et al., 2018).

Because we reasoned that the pattern of storage polysaccharide accumulation is a dynamic process intimately interconnected with those controlling tuber differentiation and growth, here we embarked on transcriptomic profiling of genes credited to affect morphological and physiological transitions throughout tuber growth and the metabolism of storage carbohydrates. To this end, the quantification of inulin was coupled to the microarray analyses on two *H. tuberosus* clones, “VR” and “K8-HS142,” which differ in their growth habits, at three tuber developmental stages: initial tuberization (T_0_), maximum elongation rate (T_3_), and physiological maturity (T_m_).

The information we acquired is crucial to breed *H. tuberosus* clones with increased levels of inulin, which in turn could be used as resources demanding low input either for biofuel production in alternative to staple crops or for the food industry.

## Materials and Methods

### Plant Materials

The *H. tuberosus* rhizomes and tubers were harvested from plants of two different clones: the multi-stem “Violet de Rennes” (“VR”), provided by the breeding station at Montpellier, INRA (Institute National de la Recherche Agronomique), France, and the mono-stem “K8-HS142” selected at the University of Tuscia, Viterbo, Italy, from the half-sib progeny of the “K8” variety selected from Germany.

### Field Experiments

Plants from the two mentioned *H. tuberosus* clones were grown in a loam soil at the Experimental Farm of the University of Tuscia, Viterbo (42°25'7''N, 12°6'15''E), Italy, from early spring, when seed tubers were sown, to the late fall of the same year, when tubers were collected.

The clones were planted in field plots arranged according to a randomized block design replicated twice. A plot density of 8 plants m^-2^ was adopted. Plants were watered at weekly interval by supplying the entire volume of water that was lost in 1 week by evapotranspiration after correction for the rainfall that occurred in that period.

### Tuber Phenophases

**Figure 1:**
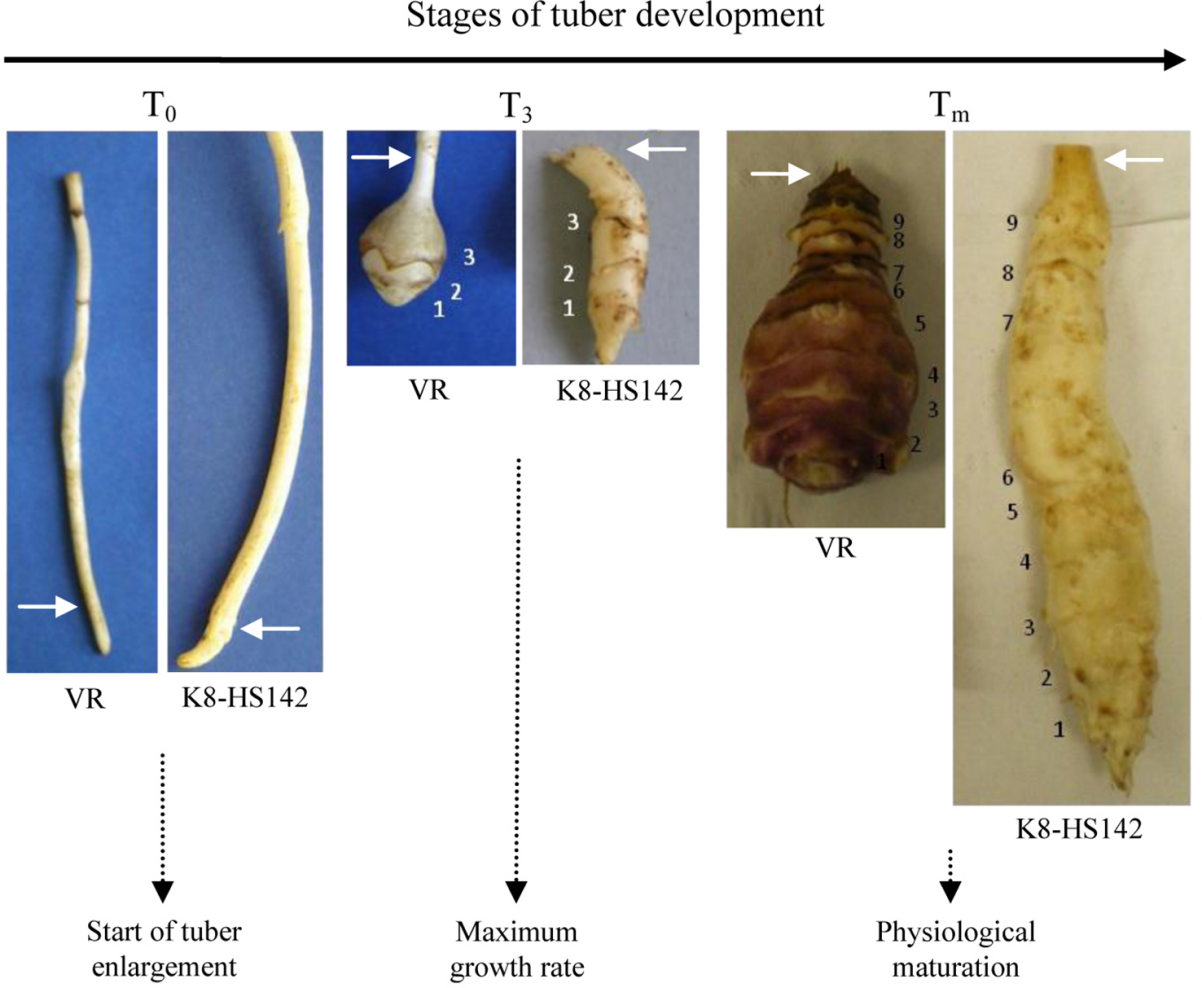
Morphological comparison of the rhizomes of *Helianthus tuberosus* “VR” and “K8HS142” clones during the tuber development. The white arrows point to the initial part of tuber enlargement. The sizes at the three stages are on scale. The number of nodes is reported.

The carbohydrate content measurement and the RNA purification were carried out from tubers harvested when they displayed the three phenophases illustrated in **Figure 1**. The length of phenophases was evaluated as the number of days from March 31, when the tubers of “VR” and “K8-HS142” were planted.

The three phenophases were identified as follows: T_0_ (pre-tuberization), T_3_ (active tuber growth), and T_m_ (tuber physiological maturity). At the T_0_ stage, the rhizomatous apex was not yet enlarged and was just going to evolve into tuber (**Figure 1**). The T_3_ stage was reached when tubers had three elongated internodes, exhibited the maximum elongation rate, and reached nearly one-third of the final length (**Figure 1**). At the T_m_ stage, tubers had nine internodes and reached the physiological maturity (no more increase in weight and length and the final epidermal coloration was expressed entirely). Tuber morphological traits, such as weight and shape, were evaluated at T_m_. The length/width ratio of the tuber was used to calculate the shape index according to Pas'ko (1973).

For RNA extraction, subsamples of the freshly harvested rhizome tips (T_0_) and tubers at T_3_ and T_m_ stages were quickly immersed in liquid nitrogen, ground to a fine powder, and stored at -80°C. Other subsamples of the fresh harvested rhizome tips and tubers at T_3_ and T_m_ stages were lyophilized and powdered for analyzing the content of fructans and starch.

Student's *t*-statistics was used to test the null hypothesis that the mean of random samples of tubers from the “VR” and “K8-HS142” clones at the same growth stage differed for a given trait only by chance events. The Statistical Package for Social Sciences (SPSS, IBM) 21.0_Ink software was used for implementing the test.

### Carbohydrate Content in Tubers

Inulin content of tubers was assessed at the three developmental stages in both “VR” and “K8-HS142” clones. Lyophilized tubers were powdered using the shredder knives SM 100 (*Retsch*). Each processed sample was collected in a glass vessel, closed air-tight with its cover, and refrigerated until analyzed for the quantitative determination of the content in free sugars, such as glucose (Glu_F_), fructose (Fru_F_), and sucrose (Suc_F_), as well as complex sugars (inulin). One gram of freeze-dried powder was mixed with 10 ml of water and boiled for 1 h. The resulting samples were then centrifuged at 2,500*g* for 5 min at room temperature (RT), and the supernatant was recovered. A fraction of the supernatant (unhydrolyzed) was diluted (1:2.5) with water and temporarily refrigerated. This fraction contained Glu_F_, Fru_F_, and Suc_F_ together with inulin. A further 2-ml fraction of supernatant was mixed with 0.5 ml of 1N HCl (pH 3.0) and boiled for 20 min to obtain acid hydrolysis of the sucrose and inulin carbohydrates. After neutralization with 0.5 ml NaOH 1N, the hydrolyzate was diluted with 4 ml of water to achieve the same 1:2.5 of supernatant-to-water volume ratio used for preparing the unhydrolyzed fraction. This fraction contained Glu_F_ and Fru_F_ only. The unhydrolyzed and hydrolyzed fractions of tuber extracts were purified according to the official method AOAC 982.14 (Cunniff, 1995) by using Sep-PAK^®^ C18 cartridges 300 mg (*Waters Corporation*, USA) and filtrated through cellulose membranes φ 0.20 μM (*Agilent Technologies*, Germany). The purified fractions were eluted through YMC-pack polyamine II VS 12 S-5 μM, 10 mm × 4 mm (*YMC*, Japan), and then used for high-performance liquid chromatography (HPLC) analyses using the Agilent 1100/1200 apparatus (*Agilent Technologies*, Germany). A volume of 0.75 ml of eluate and 2.25 ml of acetonitrile (mobile phase) were mixed to prepare the sample to be injected onto the column which was kept at 25°C during sample flow. Solutions of pure glucose, fructose, and sucrose at known concentrations (*Carlo Erba Reagenti*, Italy) were injected as controls. The content of the Glu_F_, Fru_F_, and Suc_F_ were estimated from the sugar-specific absorbance peaks obtained during HPLC analysis of the unhydrolyzed fraction of the tuber extract. Fru_F_ indicates the cumulative fructose content from both the free fructose and the free sucrose in that fraction. Glu_F_ indicates the cumulative glucose content from both the free glucose and the free sucrose in that fraction. The total glucose (Glu_T_) and fructose (Fru_T_) contents from the free and inulin carbohydrates were detected by HPLC analysis of the hydrolyzed fraction of the tuber extract. The amount of fructose released by inulin during the hydrolysis (Fru_H_) was calculated as Fru_T_ - Fru_F_ difference. The amount of glucose, Glu_H_, released by inulin during the hydrolysis, was estimated as Glu_T_ - Glu_F_ difference.

Based on the above HPLC detection of the quantity of glucose and fructose, the inulin content was estimated according to Prosky and Hoebregs (1999) and Negro et al. (2006) using the following formula: *k* · (Glu_H_ + Fru_H_), where k is a correction factor for water loss during hydrolysis whose numerical value was calculated by means of the following relationship: k=[180+162 ⋅(n−1)]÷180 n  where *n* is the average degree of polymerization, with $n=\;\frac{FruH}{GluH}+\;1$.

The difference between clones for fructan content in the tubers sampled at the three T_0_, T_3_, and T_m_ growth stages was analyzed by a one-way ANOVA analysis (*p* < 0.05).

#### Starch Content in Tubers

The Megazyme amylose/amylopectin determination kit (*Megazyme* International Ireland Ltd, Bray, Ireland) was used according to the manufacturer's instructions to detect the starch content in 25 mg of lyophilized powder of the tuber tissues collected at the T_m_ stage from both clones. The sample of the lyophilized powder was wholly dispersed by heating in dimethyl sulfoxide (DMSO). Lipids and free D-glucose were removed by precipitating the starch components in 95% ethanol and recovering the precipitate by centrifugation at 2,000*g* for 5 min. The precipitated sample was dissolved in 2 ml DMSO and placed in boiling water bath for 15 min. An aliquot of 0.5 ml of this solution was mixed with 4 ml of 100 mM sodium acetate buffer, pH 4.5. Then, 0.1 ml of amyloglucosidase/α-amylase solution was added, and the mixture was incubated at 40°C for 10 min to hydrolyze starch to D-glucose. One milliliter of this solution was mixed with 4 ml of a reagent containing glucose oxidase plus peroxidase and 4-amino antipyrine and incubated at 40°C for 20 min. The absorbance of this sample and of the D-glucose controls was read at 510 nm against the reagent blank.

### Microarray Experiment

#### Expressed Sequence Tag Library for Designing the Microarray

A set of 40,361 expressed sequence tags (ESTs) from the *Ht*_CHT(LMS)_norm library built from *H. tuberosus* tissues of different plant organs, such as seedlings, leaves, flowers, achenes, and tubers, was obtained from “The Compositae Genome Project” U.S. research consortium website^1^.

Functional annotation of the EST sequences was performed according to the Gene Ontology (GO) criteria by using tools contained in the mentioned Blast2GO bioinformatics platform.

Putative biological and molecular functions of the retrieved proteins were illustrated by the *make combined graph* function in Blast2GO. The absolute (AS) and relative (RS) scores were used for calculating the extent to which EST/gene falls within a given GO class at the hierarchical levels 6, 7, and 8. The AS score was provided by Blast2GO, while the relative score (RS) was determined by dividing the AS score related to each GO class by the sum of ASs from all the GO classes. A BlastN analysis built in the Blast2GO package (Götz et al., 2008) was performed on the abovementioned EST query sequences.

In order to elevate the accuracy of the annotation outcome, the accessions identified by the BlastN candidate hits for a given EST queried were further filtered out by discarding those ones with *E-*value >10^-60^ and sequence similarity <80% (**Figure S2**). The EST accessions identified in the final set were eventually used to retrieve protein names and related IDs from UniProt and KEGG Maps. For the purpose of this study, only genes falling within the three main categories of carbohydrate metabolism, gene expression and protein metabolism, and cell development were considered. A further set of 80 ESTs/genes encoding enzymes for fructans in *Cynara scolymus*, *Chicorium intybus*, *Allium sativum*, *Festuca arundinacea*, *Lolium perenne*, starch in *Solanum tuberosum* and *Brassica rapa*, and cell development in *Arabidopsis thaliana* were also included. An additional group of 150 different EST*s* for housekeeping genes (including genes for actin 11, actin 2/7, adenine phosphoribosyltransferase, β-actin, β-tubulin, cyclophilin, elongation factor 1α, elongation factor 1β, HSP90, polyubiquitin, ubiquitin-like, 18S rRNA, tubulin α-3/α-5 chain, tubulin β-9, rubisco, histone H4, phosphoglycerate kinase, phosphoribulokinase, plastocyanin, EIF4) from different plant species (*Prunus persica*, *Vitis vinifera*, *Triticum aestivum*, *Beta vulgaris*, *Arabidopsis thaliana*, *Coffea arabica*, *Lactuca sativa*, and others) were also retrieved from NCBI database and used to design the microarray. Features from bacterial species were included as the negative control. Globally, a set of 6,365 EST/genes, mainly including structural genes and some transcription factors related to carbohydrate metabolism and tuber development, were selected for creating the microarray platform (**Table 1**). The Cell Designer™ v4.2 software tool (Funahashi et al., 2003) was used to map the networks of molecular functions for inulin, starch, and sucrose carbohydrate metabolism (Edelman and Jefford, 1968; Geigenberger, 2011) occurring in the vacuole, amyloplast, and cytosol, respectively, and create a comprehensive scheme.

**Table 1:** Criteria pursued to select the expressed sequence tags (ESTs) spotted on the GeneChip.

CLASS		AMOUNT
A	ESTs included in the *Helianthus tuberosus* CHT(LMN)_norm expression library	39,270
A.1	ESTs annotated by Blastx tool	37,821
A.1.1	ESTs left upon filtering by using a minimum cutoff *E*-value <10^-40^ and *Sequence Similarity >*75%, of which:	16,739
A.1.1.1	Potentially involved in the biological *processes of carbohydrate metabolism*	1,227
A.1.1.2	Potentially involved in the biological *processes of gene expression and protein metabolism*	4,054
A.1.1.3	Potentially involved in the biological *processes of cell development*	3,757
B	ESTs from other plant species involved in the biological processes of carbohydrate metabolism and cell development	332
C	ESTs totally selected for designing the GeneChip	9,370
**D**	**Unigene ESTs at last selected to build probes placed onto the GeneChip**	**6,365**

The set of ESTs employed to design the microarray platform are grouped according to their origin and putative biological functions assessed upon blasting in Blast2GO.

#### Microarray Platform Production

The microarray was produced according to the MIAME guidelines^2^ by exploiting a Custom Array™ 2 × 40k platform (CombiMatrix**®**) synthesized at the University of Verona^3^, Verona, Italy, which contained 40,000 features each consisting of 30–35 *mer* oligonucleotide probes designed using the OligoArray 2.1 software. Two to four probes were designed for each of the 6,365 selected EST/genes, and each probe was replicated up to four times on the microarray.

#### RNA Isolation and Microarray Hybridization

RNA from tubers at T_0_, T_3_, and T_m_ growth stages were collected from three different biological replicates for both “VR” and “K8-HS142.” Overall, two sets of RNA, each consisting of 18 RNA preparations (three biological replicates × three stages × two clones) were isolated and processed, one for microarray and the other for qRT-PCR analyses.

Total RNA was purified from each sample starting from 500 to 800 mg of tuber tissue (previously ground in liquid nitrogen) using the TRIzol^®^ reagent (*Life Technologies*) and the procedure reported by Chomczynski and Sacchi (1987) followed by a DNase I treatment (*Ambion*) aimed to remove any DNA contamination. Purified RNA samples were controlled for their purity by using a Nanodrop 1000 spectrophotometer (*Thermo Scientific*) and integrity by means of a Bioanalyzer 2100 (*Agilent Technologies*) according to Schroeder et al. (2006) upon a proper preparation by the accompanying RNA 6000 Nano Kit (*Agilent Technologies*).

The RNA ampULSe: Amplification and Labeling Kit (with Cy5 for CombiMatrix arrays) (*Kreatech*) was used according to manufacturer's instructions to synthesize and clean cDNA and antisense RNA (aRNA), labeling the aRNA with the fluorochrome cyanine Cy5, and to accomplish the fragmentation of labeled aRNA. The microarray was hybridized with 3 μg of labeled aRNA for 16 h at 45°C, then washed using serial washings based on cleaning solutions, such as 6X SSPET, 3X SSPET, 0.5X SSPET, PBST, PBS, and eventually imaged. Each aRNA used for microarray hybridization represented a “C_i_” condition of hybridization.

#### Data Acquisition and Analysis

Using a PerkinElmer 400XL scanner (*PerkinElmer*), Tagged Image File Format (TIFF) images of the gene spots were obtained, exported to the Microarray Imager 5.8 (*CombiMatrix*) software for densitometric analysis and imported in LIMMA statistical package (Smith, 2004) by the use of “Read.maimages” function.

The signal observed from a microarray spot resulted from the combination of the true foreground emission from the specific hybridization event and the background emission due to nonspecific hybridization. Therefore, using the “BackgroundCorrect” function, the signals rising from contaminations and imperfections, such as irregular contour, donut shapes, artifacts, and low or heterogeneous expression, were removed, and the accuracy of spot-signal measurement was ameliorated.

To achieve a meaningful comparison of the signals from different biological*/*technical replicates, the set of emission signal intensities from different arrays were normalized.

The variation across arrays due to sample preparations and array manufacturing and processing (labeling, hybridization, and scanning) was smoothed, adopting the quantile normalization algorithm implemented in the “NormalizeBetweenArrays” function.

With the aim to detect the set of DE EST/genes, the emission signals were analyzed using the core component of the LIMMA package to fit gene-wise linear models on gene expression data. DE was assessed by estimating log intensities for each probe hybridized to the target aRNA samples.

The “model.matrix” function was used to set a matrix of expression levels based on the emission signal intensity from each probe. The different probes were placed in the matrix rows, and the different conditions analyzed in the experiment were allocated to the columns of the matrix. The row-wise probe emission signal intensities were used for linear regression analysis using the “lmFit” function.

Once a linear model was fitted, log_2_-fold changes of the emission signal intensities on the entire set of a possible pair of C_i_ conditions for each probe were computed using the “makeContrast” function.

Differentially expressed (DE) ESTs were identified as the ones whose corresponding expression value ratios in the C_1_ vs. C_2_ conditions showed at least a two-fold change (FC ≥2). The moderated *t*-statistics was used to assess the significance of the log_2_-fold changes of the emission signal intensities for that pair of C_i_ conditions for a given probe/EST/gene. The moderated *t*-statistic has the same interpretation as an ordinary *t*-statistics except that the standard errors have been moderated across genes, squeezed toward a common value, using a simple Bayesian model.

The C_1_ and C_2_ conditions were considered significantly different in gene expression levels when log_2_│I_1_ I_2_^-1^│≥ 1 and *p*_adj_ for the moderated *t*-statistics <0.05, I_1_ and I_2_ representing the mean value of the emission signal intensity from the probe/EST/gene hybridized with the labeled aRNA-Cy5 samples that made the C_1_ and C_2_ conditions, respectively.

A one-way ANOVA (*p* < 0.05) approach was adopted to compare the significance of the emittance differences over all the three growth stages. To sort among DE EST/genes clustered into a specific GO level (=8) those that likely play a more relevant role in either carbohydrate metabolism or tuber growth, the DE EST/genes were then blasted against UNIPROT and KEGG databases.

The data reported in this study have been deposited in NCBI's Gene Expression Omnibus (Edgar et al., 2002) and are accessible through GEO Series accession number GSE132955 (https://www.ncbi.nlm.nih.gov/geo/query/acc.cgi?acc=GSE132955).

### Quantitative Real-Time PCR Validations

To validate the differential gene expression values obtained by microarray analysis, quantitative real-time PCR (qRT-PCR) analyses were performed on a subset of four key genes implied in the hexose metabolism [*sucrose synthase (SuS)*, *phosphoglucomutase (PGM*)] and fructan (*1-SST*, *1-FFT*) biosyntheses. The target genes were among those predicted by microarray analysis to be DE among the three different tuber developmental stages.

A primer pair specific to each target gene was designed using the “OligoExpress” software (*Applera Biosystems)*. Forward and/or reverse primers were designed on the probe emitting the highest hybridization signal from the set of features from a given EST (**Table S1**).

Three RNA samples for each condition were used for the synthesis of cDNA. For each sample, 3 μg of RNA were reverse transcribed in duplicate using the SuperScript III H-Reverse Transcriptase (*Life Technologies*) and 100 pmol of random hexamers (*Pharmacia Biotech*) according to the supplier's instructions. The two replicated reactions were then mixed.

The successful synthesis of cDNA was tested by PCR amplification with 18S and 26S external transcribed spacer (ETS associated with 18S and 26S rDNA) (**Table S1**). The 25-μl PCR mixture contained 2.5 mM MgCl_2_, 0.2 mM of each dNTPs, 10 nM of each primer, and 1 U of Taq DNA polymerase (*Euroclone*). PCR reactions were performed in a 9700 PCR system (*Life Technologies*), adopting the following thermal profile: 94°C, 2 min followed by 50 cycles of denaturation at 94°C, 5 s, annealing at 60°C, 10 s and extension at 72°C, 30 s, with a final extension at 72°C, 5 min.

An aliquot of 3 μl of 1:10 diluted mixture of cDNA was used in the PCR reaction, which was made up using the Power SYBR-Green PCR core mix (*Applied Biosystems*) according to the supplier's instructions in 20 μl final volume in the presence of 2.5 pmol of each primer. Four replicates were analyzed for each gene tested in the 18 conditions. Cycling parameters were two initial steps at 50°C, 2 min, and 95°C, 2 min, then 50 cycles each including a step at 95°C, 15 s and a step at 60°C, 1 min, and a final step at 60°C, 10 min. Afterward, the dissociation protocol was performed.

Amplifications were performed into an ABI PRISM 7300 SDS apparatus (*Applied Biosystems*). For each gene, the average threshold cycle (C_t_) was determined. Standard curves for target genes and the housekeeping gene encoding for the elongation factor-1α (*EF-1α*), employed as internal control, were obtained by the amplification of a serially diluted mixture of cDNA samples, with six dilution points, each one replicated four times to calculate the amplification efficiency of each primer pair. The gene expression quantification method (2^**^**ΔCt^) based on the differences between the relative expression of the target and the reference *EF-1α* gene was adopted in order to compare the relative expression profiles among genes and clones as reported in Passeri et al. (2017).

Significance of the differences between mean values for the relative expression detected by the RT-qPCR for the two clones at the three tuber developmental stages were analyzed by a two-way ANOVA (*p* < 0.05) procedure embedded in the *R* statistical package.

## Results

### Morphological and Physiological Differences Between “VR” and “K8-HS142” Plants and Tubers

**Figure 2:**
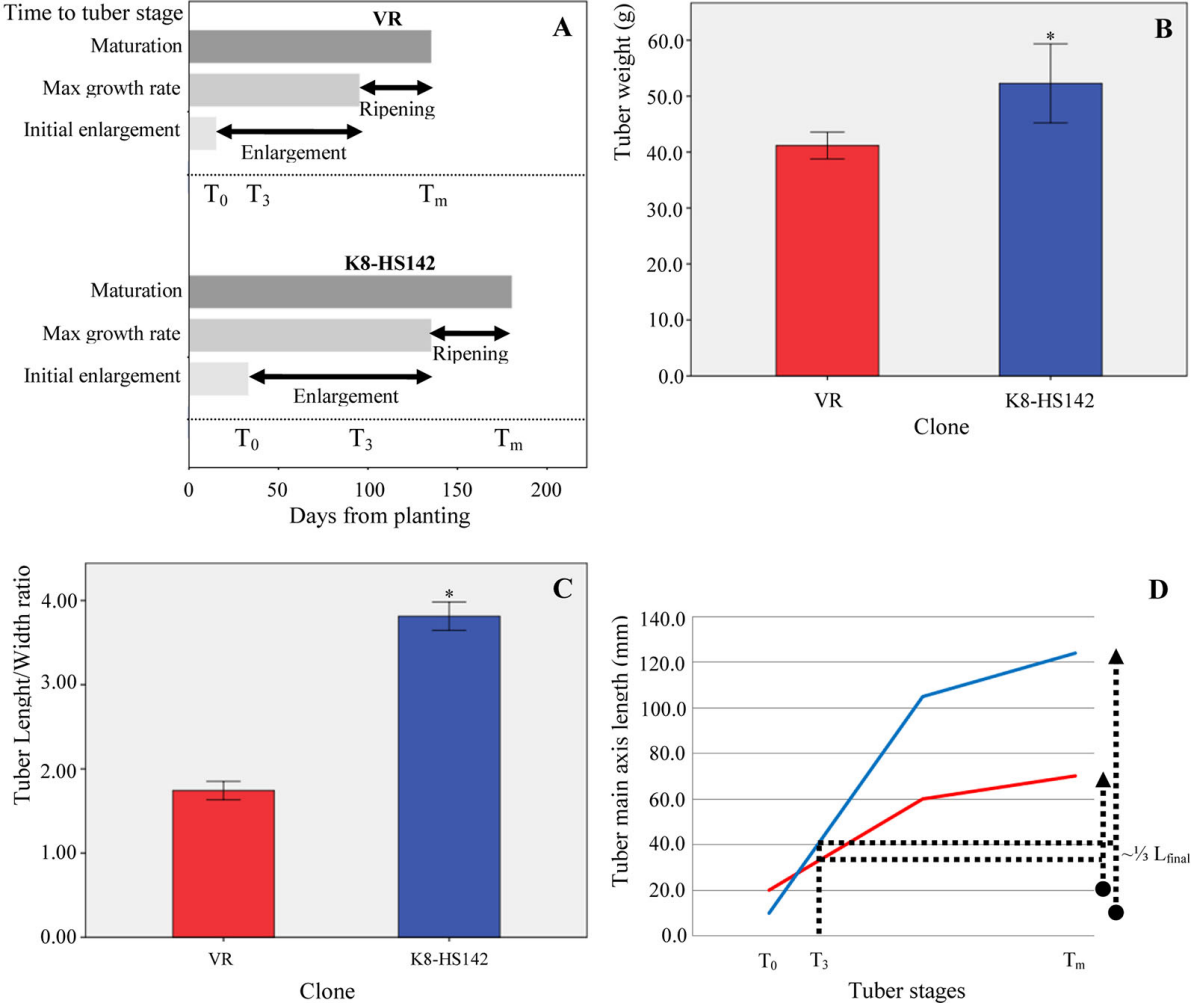
Tuber traits in *Helianthus tuberosus* “VR” and “K8-HS142” clones. **(A)** *Phases of tuber development*. Bars show the duration of each of the three tuber developmental stages, from the onset of tuberization to physiological maturation. The scale starts from the day of planting. Data are from the average of three plants *per* clone. **(B)** *Tuber weight*. Data are from tubers collected from four different plants *per* clone. **(C)** *Tuber shape*. Ratio between length and width in tubers of “VR” and “K8-HS142” clones, collected from four different plants. According to Pas'ko (1973) tubers from “VR” and “K8-HS142” were classified as short pear-shaped and spindle-shaped, respectively (**Figure 1**; **Figure S1**). **(D)** Temporal variation in the length of the main axis (mm) of “VR” (red line) and “K8-HS142” (blue line) tubers. *: Significances (*p* < 0.05). The bar indicates ±1 standard error.

Both in “VR” and “K8-HS142” clones, the main stem raised directly from the seed tuber. However, “VR” plants displayed a branched architecture due to the sprouting of the lateral buds from basal nodes on the main stem (**Figure S1-A1**), whereas the most “K8-HS142” plants were unbranched (**Figure S1-B1**). The rhizome was shorter in “K8-HS142” than in “VR,” determining denser biomass around the crown area in the tubers of former than of the latter genotype (**Figures S1-A2, B2**). The number of days from tuber planting to the beginning of flowering varied between about 120 days in “K8-HS142” (flowering during the first half of July) and 190 days in “VR” (flowering at the end of September). As for tuber development, “VR” reached T_0_ in the middle of June, about 20 days earlier than “K8-HS142” and reached the T_3_ stage about 40 days earlier than “K8-HS142.” The T_m_ stage, when tuber ripening ended, occurred at early October in “VR,” about 60 days earlier than “K8-HS142,” but the length of the ripening period was similar between the clones (**Figure 2A**).

At T_m_, the fresh tuber weight and the length/width ratio were significantly higher in “K8-HS142” than in “VR” (52.27 ± 14.12 g vs. 41.17 ± 4.79 g and 3.81 ± 0.34 vs. 1.74 ± 0.22, respectively) (**Figures 2B, C**).

From the onset of tuberization to maturation, the main axis length in “VR” and “K8-HS142” increased up to nearly 50 and 115 mm, respectively (**Figure 2D**).

Based upon these data, the tubers of “VR” and “K8-HS142” were classified as pear- and spindle-shaped (**Figure 1**; **Figures S1-A2, B2**), respectively, according to Pas'ko (1973).

### The Concentration of Storage Polysaccharides in Developing Tubers

**Figure 3:**
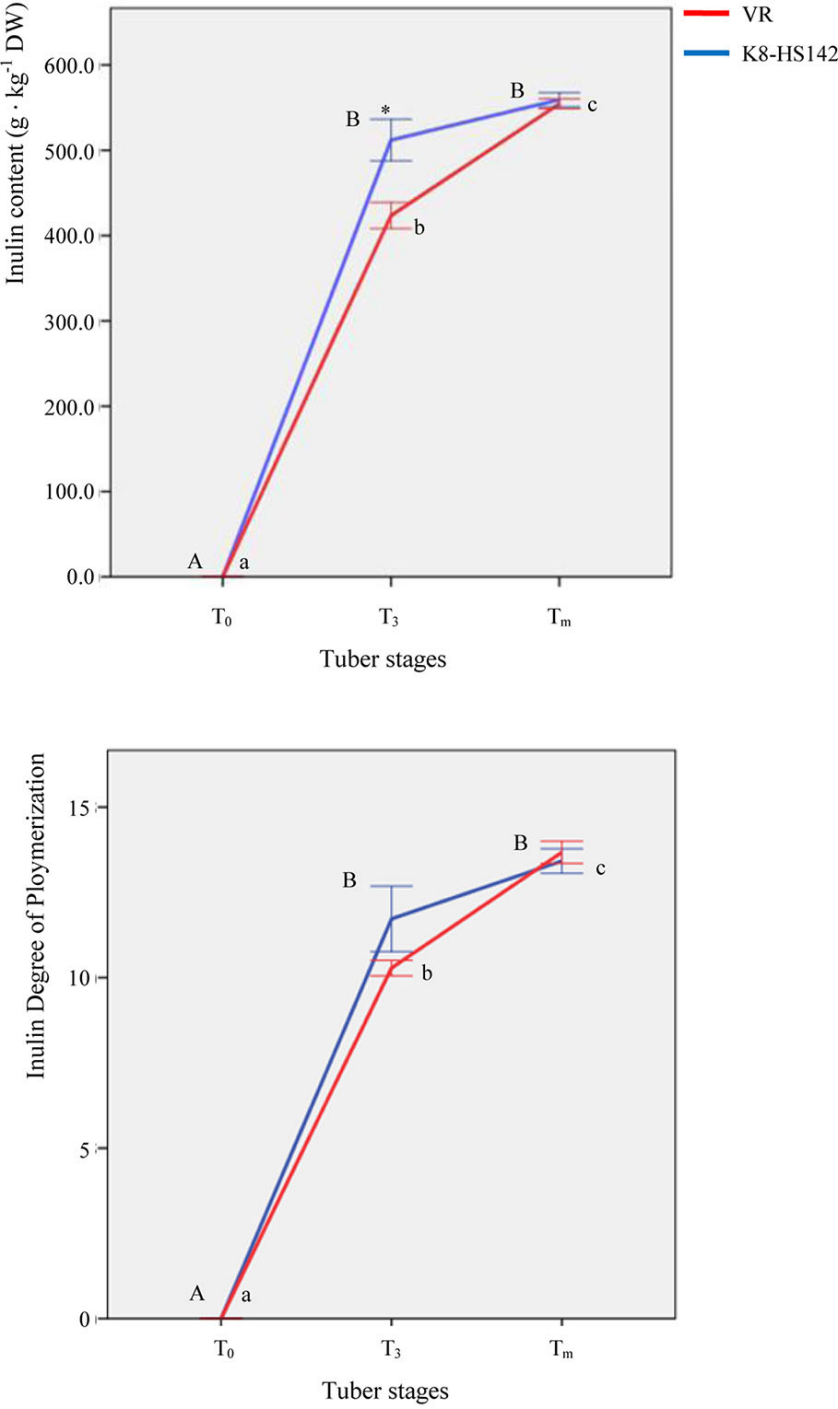
Inulin content and degree of polymerization (DP) in tubers of *Helianthus tuberosus* “VR” and “K8-HS142” clones. Significantly different values (*p* < 0.05) at different stages in the same clone are labeled with different letters (lowercase for “VR,” uppercase for “K8-HS142”) and those between clones at the same stage with *. The bar indicates ±1 standard error.

The variation in the content of the main complex carbohydrates was analyzed in developing tubers. The inulin was undetectable in the rhizome tips of both clones at T_0_. It increased sharply at T_3_, when “K8-HS142” exhibited a significantly higher content (512.03 ± 24.43 g · kg^-1^ DW) compared to “VR” (423.49 ± 15.31 g · kg^-1^ DW). At T_m_, the increment of inulin was significant only in “VR,” and both clones had a similar inulin content: 559.32 ± 8.18 (“K8-HS142”) and 555.53 ± 5.78 (“VR”) g · kg^-1^ (**Figure 3**).

The degree of polymerization basically followed the pattern of total inulin content: at T_3_, the DP was higher in “K8-HS142” (11.72 ± 0.96) than “VR” (10.28 ± 0.23), whereas at T_m_, the DP increased in “VR” (13.67 ± 0.33) but not significantly in “K8-HS142” (13.42 ± 0.36) (**Figure 3**).

The starch content expressed as percentage of lyophilized tuber weight at T_m_ was 0.31% ± 0.02% in “VR” and 0.64% ± 0.18% in “K8-HS142”. Therefore, the starch content of tubers at the physiological maturity was insignificant.

### Expression Analysis of the Set of Expressed Sequence Tags/Genes on the Microarray Chip

Out of the 6,365 ESTs spotted, significant differential expression between “VR” and “K8-HS142” was detected for 60 and 11 EST/genes at T_0_ and T_3_ stages, respectively (**Table 2**), whereas no significant differences were detected between clones at tuber maturity (T_m_).

**Table 2:** Number of differentially expressed (DE)/expressed sequence tags (ESTs).

Case	Experimental conditions		Total number of DE/ESTs
A	“VR” against “K8-HS142”	T_0_	60
		T_3_	11
		T_m_	0
B	“VR” and “K8-HS142”	T_0_ vs. T_3_	11
		T_0_ vs. T_m_	79
		T_3_ vs. T_m_	3
C_1_	“VR”	T_0_ vs. T_3_	23
		T_0_ vs. T_m_	654
		T_3_ vs. T_m_	697
C_2_	“K8-HS142”	T_0_ vs. T_3_	97
		T_0_ vs. T_m_	501
		T_3_ vs. T_m_	612

The total number of the DE ESTs are reported for different cases of comparisons: between “VR” and “K8-HS142” clones at each tuber stage (A), between tuber stages but shared by the two clones (B), and between tuber stages in each of the two “VR” (C_1_) and “K8-HS142” (C_2_) clones.

Within clones, the highest number of DE ESTs (DE/ESTs) occurred when compared T_3_ vs. T_m_ for both “VR” (697) and “K8-HS142” (612), whereas the lowest in T_0_ vs. T_3_, being 23 and 97 in “VR” and “K8-HS142,” respectively (**Table 2**). Despite this high number of DE ESTs, those shared between the clones across the different developmental stages were only a few between T_0_ and T_3_ (11) and three between T_3_ and T_m_ (**Table 2**), whereas the highest number occurred between T_0_ and T_m_ (79).

#### Differentially Expressed/Expressed Sequence Tags Between “VR” and “K8-HS142” Clones at T_0_ and T_3_

Among the 60 DE/ESTs between clones at T_0_, 8, 26, and 26 were sorted into the macrocategories carbohydrate metabolisms, gene expression/protein metabolism, and cell development, respectively (**Table S2**). The categories of GO analysis comprised terms such as “Glucose metabolic P,” “Hexose catabolic P,” “Cellular protein Catabolic P,” and “Organ development P,” just to cite a few. Then, in order to focus on ESTs intimately connected to the processes of carbohydrate metabolism and tuber growth, 32 DE/ESTs that yielded a significant match with proteins related to these functions as per UNiProt and KeGG analyses were specifically considered (**Tables 3**–**5** and references therein).

**Table 3:** Differentially expressed (DE)/expressed sequence tags (ESTs) between clones at T_0_ taking a role in the carbohydrate metabolism.

EST ID	Enzyme			Gene expression profile			**References**
	UNIPROT name	KEGG code	GO Biol. Proc.	↑	FC	*p*	
*CHTM20084.b1* *H05.ab1*	Enolase	EC:4.2.1.11	Canonical glycolysis	K8-HS142	1.21	*	Van der Straeten et al. (1991)
*CHTM25255.b1* *N02.ab1*	2,3-bis-phosphoglycerate mutase	EC:5.4.2.4	Canonical glycolysis	K8-HS142	1.40	*	Mazarei et al. (2003)
*CHTM27988.b1* *H14.ab1*	Phospho-fructokinase α subunit	EC:2.7.1.11	Canonical glycolysis	K8-HS142	2.47	**	Theodorou et al. (1992)
*gi_31324479 gb_CA513537*				VR	1.29	*	
*CHTM6350.b1* *K04.ab1*	NADH dehydrogenase	EC:1.6.5.3	Electron transport chain	K8-HS142	1.07	**	Douce et al. (1973)

Gene code (EST ID), name of the coded protein according to UNIPROT, the related KEGG code, and the specific Gene Ontology (GO) classification are reported for the DE ESTs involved in the “Carbohydrate metabolic process” (GO ID:0005975).

The fold change (FC) is a log_2_ value of the ratio between the levels of emittance of ESTs in “VR” and “K8-HS142.” The sign **↑** refers to an overexpressed EST.

One, two and three asterisks correspond to 0.01 ≤ p < 0.05, two (**) to 0.001 ≤ p < 0.01 and three (***) to p < 0.001, respectively.

A proper publication reference accompanies each DE/EST, focusing the gene function.

**Table 4:** DE/ESTs between clones at T_0_ taking a role in cell development.

EST ID	Enzyme			Gene expression profile			References
	UNIPROT name	KEGG code	GO Biol. Proc.	↑	FC	*p*	
*CHTS9981.b1* *J24.ab1*	Myo-inositol-1-phosphate synthase	EC:5.5.1.4	Cell growth	K8-HS142	2.60	*	Nunes et al. (2006)
*CHTM12531.b1* *E14.ab1*				K8-HS142	2.04	*	
*CHTM9961.b1* *B20.ab1*				K8-HS142	1.55	*	
*CHTS18875.b1* *E15.ab1*	Non-ATPase subunit 26S proteasome	–	Cell proliferation	K8-HS142	1.49	**	Smalle and Viestra (2004)
*CHTM27779.b1* *F09.ab1*	Ubiquitin-like modifier- activating protein	–	Cell cycle process	K8-HS142	1.59	*	Streich and Lima (2014)
*CHTM21337.b1* *A08.ab1*	Shaggy-related protein kinase ϵ protein	EC:2.7.11.1	Cell differentiation	K8-HS142	2.24	*	Pérez-Pérez et al. (2002)
*CHTM19297.b1* *B01.ab1*	ι protein			K8-HS142	1.83	**	
*CHTM19242.b1* *C11.ab1*	η protein			K8-HS142	1.34	*	
*CHTS10787.b1* *E09.ab1*	Auxin response factor 2	EC:6.13.2.19	Cell division	VR	1.64	*	Wen et al. (2014)
*CHTS19020.b1* *G04.ab1*	E3 ubiquitin-protein ligase RGLG2	–	Root development	VR	1.40	*	Schruff et al. (2006)

ESTs affecting “Cellular process” (GO ID: 0009987). Legend as in **Table 3**.

**Table 5:** Differentially expressed (DE)/expressed sequence tags (ESTs) between clones at T_0_ taking a role in the metabolism of nucleic acids and proteins.

EST ID	Enzyme			Gene expression profile			References
	UNIPROT name	KEGG code	GO Biol. Proc.	↑	FC	*p*	
*CHTM7783.b1 N01.ab1*	S-adenosyl-l-homocysteine hydrolase	EC:3.3.1.1	DNA methylation	K8-HS142	1.56	*	Tanaka et al. (1997)
*CHTS8157.b2* *I23.ab1*				K8-HS142	1.21	*	
*CHTM25241.b1* *A24.ab1*	E3 ubiquitin-protein ligase UPL3-like	EC:6.3.2.19	Chromatin silencing	K8-HS142	1.44	**	Song et al. (2007)
*CHTS16325.b1* *I02.ab1*	Ketol-acid reductoisomerase	EC:1.1.1.86	Cellular amino acid biosynthetic process	K8-HS142	1.19	*	Lee et al. (2005)
*CHTS8905.b3* *A19.ab1*	5-enol-pyruvylshikimate-phosphate synthase	EC:2.5.1.19		K8-HS142	1.15	*	Stauffer et al. (2001)
*CHTM5296.b1* *P04.ab1*	Tyrosyl-tRNA synthase	EC:6.1.1.1	Translation	K8-HS142	2.12	*	Bick et al. (1970)
*CHTM2921.b1* *A12.ab1*	Glycyl-tRNA synthase	EC:6.1.1.14		K8-HS142	1.50	*	Uwer et al. (1998)
*CHTM8693.b1* *I14.ab1*	Glutamynil-tRNA synthase	EC:6.1.1.17		K8-HS142	2.16	***	Siatecka et al. (1998)
*CHTM19206.b1* *K01.ab1*	Initiation factor 4A	EC:3.4.22.44		K8-HS142	1.63	*	Mandel et al. (1995)
*CHTS12242.b1* *D14.ab1*				K8-HS142	1.61	*	
*CHTM25642.b1* *D04.ab1*				K8-HS142	1.39	*	
*CHTS12252.b1* *H16.ab1*	Elongation factor 1α	EC:2.7.7.4		VR	2.78	**	Moore et al. (1998)
*CHTM11911.b1* *M01.ab1*	Heat shock protein 70	EC:1.3.1.74	Protein stabilization	K8-HS142	2.56	**	Lee and Vierling (2000)
*CHTL2359.b2* *M13.ab1*				K8-HS142	1.82	*	
*CHTM20288.b1* *P08.ab1*	UDP-glucose:glycoprotein glucosyltransferase	EC:2.4.1.-	Protein folding	K8-HS142	1.15	*	Arnold et al. (2000)
*CHTS11460.b1* *H10.ab1*	Proteasome subunit beta type-5A	EC:3.4.25.1	Protein depolymerization	VR	1.03	*	Moon et al. (2004)
*CHTM12018.b1* *D05.ab1*	Aspartic protease	EC:3.4.23.20		VR	1.61	**	Simões and Faro (2004)

ESTs implied in the "DNA metabolic processes" (GO ID: 0006259) are shadowed in gray^+^, those in "Metabolic processes" (GO ID: 0016070) in gray^++^, those in ''Organo-nitrogen compound metabolic process" (GO ID: 1901564) in gray^+++^, and those in "Protein metabolic processes" (GO ID: 0019538) in gray^++++^. Legend as in **Table 3**.

Out of the five ESTs involved in carbohydrate metabolism, four were upregulated in “K8-HS142”: *CHTM20084.b1*, encoding for enolase; *CHTM25255.b1* for 2,3-bisphoglyceratemutase implied in the canonical glycolysis; *CHTM6350.b1*, encoding for NADH dehydrogenase, a crucial enzyme for the electron transport chain in the mitochondrial membrane; and an EST native from *H. tuberosus* (*CHTM27988.1*) coding an α-subunit of phosphofructokinase. The EST for an α-subunit of phosphofructokinase native from *S. tuberosum* (*CA513537*) was conversely upregulated in “VR” (**Table 3**).

Concerning DE/ESTs for cellular processes, the vast majority were upregulated in “K8-HS142,” among them, those implied in cell cycle process (*CHTM27779.b1*), cell differentiation (*CHTM19297.b1*, *CHTM21337.b1*, and *CHTM19242.b1*), cell proliferation (*HTS18875.b1*), and cell growth (*CHTM12531.b1*, *CHTS9981.b1*, and *CHTM9961*). Only two, *CHTS10787.b1* and *CHTS19020.b1*, strictly associated to cell division and cell root development, respectively, were upregulated in “VR” (**Table 4**). Most of the genes related to gene expression/protein stabilization and folding were upregulated in “K8-HS142” (**Table 5**). These included ESTs related to: a) translational events, b) aminoacidic biosynthesis, c) protein stabilization, d) protein folding and modification, and e) epigenetic events. Conversly, ESTs related to protein depolymerization were overexpressed in “VR” (**Table 5**).

Eleven were the DE/ESTs between clones at T_3_ (**Table S3**), 10 of them categorized into the macrobiological processes “Primary metabolic P” within which an EST related to carbohydrate biosynthesis and one to protein modification-phosphorylation were upregulated in “VR” and “K8-HS142,” respectively. The remaining ESTs were upregulated either in “K8-HS142” (3) or in “VR” (5). The last DE/EST, upregulated in “K8-HS142,” is involved in cell wall modification. Anyway, after blasting in NCBI database, none of them was really credited to affect carbohydrate metabolism and storage and tuber development.

#### Differentially Expressed/Expressed Sequence Tags Across the Different Stages of *H. tuberosus* Tubers

To depict genes most likely associated to stage transition in the tubers of *H. tuberosus*, DE/ESTs across the three developmental stages were searched by merging the data from “VR” and “K8-HS142” clones.

Seventy-nine ESTs were differentially expressed between the two most extreme stages (T_0_ and T_m_) (**Table 2**) and were relative to “Cellular P” such as “Organ and shoot development” (33), “Primary metabolic P” such as “Monosaccharide metabolic P” (18), “Cellular protein metabolic and modification P,” “Nucleic acid metabolic P,” (23), and “Transport P” (4) (**Table S4**). Within the “Cellular P” and “Transport P” groups, the majority of the ESTs (20 and 4, respectively) were upregulated at T_0_, whereas in the second and third groups, the most of them peaked at T_m_ (11 and 11, respectively).

More in details, ESTs involved in basic biological processes, such as electron transport, glycolysis, aminoacid biosynthesis, cell growth, cell wall assembly and modification, water entrance into the cell and vacuole, and auxin transport were upregulated at T_m._ (**Tables 6**–**8** and references therein). Conversely, ESTs/genes involved in chromatin silencing, translation, and apoptosis together with ESTs involved in the glycolytic process, such as those for aldolase and phospho-glucomutase, were upregulated at T_m_.

**Table 6:** Differentially expressed (DE)/expressed sequence tags (ESTs) between tuber stages taking a role in the carbohydrate metabolism and sugar transport.

EST ID	Enzyme			Gene expression profile					References
	UNIPROT name	KEGG code	GO Biol. Proc.	Contest	↑	FC	p	DE source	
CHTS9987.b2 E01.ab1	Pyruvatekinase β subunit 1	EC:2.7.1.40	Canonical glycolysis	T_0_ vs. T_3_	T_0_	1.21	*	K8-HS142	Miller and Evans (1957)
				T_0_ vs. T_m_	T_0_	2.45	***	Both clones	
gi_111145720 gb_EE258072.1	Phospho-glycerate kinase	EC:2.7.2.3		T_0_ vs. T_3_	T_3_	1.62	**	K8-HS142	McMorrow and Bradbeer (1990)
				T_0_ vs. T_m_	T_m_	1.05	**	K8-HS142	
CHTM8761.b1 B08.ab1	Phospho- glucomutase	EC:5.4.2.2		T_0_ vs. T_m_	T_m_	1.21	***	Both clones	Fernie et al. (2000)
				T_3_ vs. T_m_	T_m_	1.60	***	Both clones	
CHTM19988.b1 G05.ab1	Aldolase	EC:4.1.2.13		T_0_ vs. T_m_	T_m_	1.14	**	Both clones	Haake et al. (1998)
CHTS11266.b1 D09.ab1	NADH dehydrogenase	EC:1.6.5.3	Electron transport chain	T_0_ vs. T_m_	T_0_	1.56	***	Both clones	Douce et al. (1973)
CHTS9275.b2 E15.ab1	Sucrose synthase	EC:2.4.1.13	Sucrose biosynthetic process	T_0_ vs. T_m_	T_0_	1.86	**	Both clones	Chourey et al. (1998)
				T_3_ vs. T_m_	T_3_	1.45	*	Both clones	
CHTM3989.b2 J13.ab1	Alkaline/neutral invertase	EC:3.2.1.26	Sucrose catabolic process	T_0_ vs. T_m_	T_m_	1.05	***	K8-HS142	Sturm (1999)
gi_3367710 emb_AJ009756.1	Sucrose:sucrose 1-fructosyl-transferase	EC:2.4.1.99	Inulin biosynthetic process	T_0_ vs. T_m_	T_0_	3.83	***	Both clones	Lüscher et al. (1996)
				T_3_ vs. T_m_	T_3_	5.28	***	Both clones	
gi_3367689 emb_AJ009756.1	Fructan:fructan 1-fructosyl-transferase	EC:2.4.1.100		T_0_ vs. T_3_	T_3_	1.45	*	K8-HS142	
				T_0_ vs. T_m_	T_m_	1.47	*	Both clones	
CHTM14126.b1 L04.ab1	Sugar transporter ERD 6-like 6	EC:1.3.1.74	Sugar transmembrane transporter activity	T_0_ vs. T_m_	T_m_	1.60	***	K8-HS142	Poschet et al. (2011)
CHTM16917.b1 I05.ab1				T_0_ vs. T_m_	T_m_	1.20	***	Both clones	
CHTM25693.b1 J16.ab1				T_0_ vs. T_m_	T_0_	1.15	**	K8-HS142	

ESTs implied in the “Carbohydrate metabolic processes” (GO ID:0005975) are shadowed in gray+, those in “Transport” (GO ID:0006810) in gray^++^. DE source indicates the clone/s contributing to the DE condition. Legend as in **Table 3**.

**Table 7:** Differentially expressed (DE)/expressed sequence tags (ESTs) between tuber stages taking a role in cell development.

EST ID	Enzyme			Gene expression profile					References
	UNIPROT name	KEGG code	GO Biol. Proc.	Contest	↑	FC	p	DE source	
CHTS18557.b1 J07.ab1	UDP-galactose transporter 3	–	Cell wall assembly	T_0_ vs. T_3_	T_0_	1.04	*	K8-HS142	Norambuena et al. (2002)
				T_0_ vs. T_m_	T_0_	1.47	***	Both clones	
CHTM21118.b1 L24.ab1				T_0_ vs. T_m_	T_0_	1.14	***	Both clones	
CHTS12971.b1 F04.ab1	β-1,4-glucan synthase	EC:2.4.1.12		T_0_ vs. T_m_	T_0_	1.07	***	K8-HS142	Buckeridge (2010)
CHTM25460.b1 H05.ab1	MAP3K ε protein kinase	EC:2.7.11.25	Cell growth	T_0_ vs. T_m_	T_0_	1.42	**	K8-HS142	Suarez Rodriguez et al. (2010)
CHTM2635.b1 F12.ab1				T_0_ vs. T_m_	T_0_	1.73	***	Both clones	
CHTM2566.b1 K18.ab1	Cryptochrome 1 protein 2	–		T_0_ vs. T_m_	T_m_	1.07	***	K8-HS142	Lin et al. (1996)
CHTM18141.b1 I23.ab1	Zinc finger CCCH transcription factor	–	Uni dimensional cell growth	T_0_ vs. T_m_	T_0_	1.44	***	Both clones	Wang et al. (2008)
CHTM1800.b1 O18.ab1	Membrane steroid-binding protein 2	EC:4.1.99.3		T_0_ vs. T_m_	T_m_	1.39	**	K8-HS142	Yang et al. (2005)
CHTS11585.b1 A17.ab1	Pectin methylesterase	EC:3.1.1.11	Cell wall modification	T_0_ vs. T_m_	T_0_	1.14	***	Both clones	Micheli (2001)
CHTS9186.b3 D18.ab1				T_0_ vs. T_m_	T_0_	1.12	***	K8-HS142	
CHTM9629.b1 I07.ab1	Non-ATPase subunit 26S proteasome	–	Cell wall organization	T_0_ vs. T_m_	T_0_	1.02	**	VR	Smalle and Viestra (2004)
CHTS9721.b1 B07.ab1	Polyubiquitin 2 protein	–	Apoptotic process	T_0_ vs. T_m_	T_m_	1.26	*	VR	Zhong et al. (2005)
CHTS12298.b1 C03.ab1	Water channel protein	–	Water channel activity	T_0_ vs. T_m_	T_0_	1.05	***	VR	Chrispeels and Maurel (1994)
CHTM25693.b1 J16.ab1				T_0_ vs. T_m_	T_0_	1.15	**	K8-HS142	
CHTS15962.b1 C08.ab1	Tonoplast intrinsic protein	–		T_0_ vs. T_m_	T_0_	1.44	**	Both clones	Ludevid et al. (1992)
CHTM4514.b1 D02.ab1	Auxin influx carrier protein	–	Auxin transport	T_0_ vs. T_m_	T_0_	1.13	***	VR	Parry et al. (2001)

ESTs implied in “Cellular processes” (GO ID:0009987) are shadowed in gray^+^, those in “Water transport” (GO ID:0006833) in gray^++^, those in “Hormone transport” (GO ID:0009914) in gray^+++^. DE source indicates the clone/s contributing to the DE condition. Legend as in **Table 3**.

**Table 8:** Differentially expressed (DE)/expressed sequence tags (ESTs) between tuber stages taking a role the metabolism of nucleic acids and proteins.

**EST ID**	Enzyme			Gene expression profile					References
	UNIPROT name	KEGG code	GO Biol. Proc.	Contest	↑	FC	p	DE source
CHTS12733.b1 I15.ab1	Histone deacetylase	EC:3.5.1.98	Chromatin silencing	T_0_ vs. T_m_	T_m_	1.17	***	Both clones	Probst et al. (2004)
CHTM11351.b1 M06.ab1	GAI-like protein 1	–	Cellular amino acid biosynthetic process	T_0_ vs. T_3_	T_0_	1.02	*	K8-HS142	Fleck and Harberd (2002)
CHTM6612.b1 G21.ab1	Initiation factor 1A	EC:3.4.22.44	Translation	T_0_ vs. T_m_	T_m_	1.08	*	VR	Gross and Kinzy (2005)
CHTM1887.b1 N16.ab1	Initiation factor 4A			T_0_ vs. T_m_	T_m_	1.20	***	Both clones	Mandel et al. (1995)
CHTS15763.b1 E05.ab1	Initiation factor 5A			T_0_ vs. T_m_	T_m_	1.07	***	K8-HS142	Thompson et al. (2004)
CHTS13974.b1 L13.ab1	Calnexin homolog protein	–	Protein modification process	T_0_ vs. T_m_	T_0_	1.22	*	Both clones	Boyce et al. (1994)
CHTS14727.b1 N09.ab1				T_0_ vs. T_m_	T_0_	1.84	*	K8-HS142	
CHTS9499.b2 E24.ab1	Luminal binding protein	–		T_0_ vs. T_m_	T_0_	1.24	*	K8-HS142	Denecke et al. (1991)
CHTS14889.b1 B04.ab1				T_0_ vs. T_m_	T_0_	1.31	*	K8-HS142	

ESTs implied in the “RNA metabolic process” (GO ID:0016070) are shadowed in gray^+^, those in “Organo-nitrogen compound metabolic process” (GO ID:1901564) in gray^++^, those in “Protein metabolic processes” (GO ID:0019538) in gray^+++^. DE source indicates the clone/s contributing to the DE condition. Legend as in **Table 3**.

Two different ESTs encoding for a sugar transporter ERD 6-like 6 (*CHTM14126.b1* and *CHTM16917.b1*), driving the simple sugars movement through vacuole tonoplast, were upregulated at T_m_, while another EST for a sugar transporter ERD 6 (*CHTM25693.b1*) was upregulated at T_0_ even though at a lower intensity.

In the transition between T_0_ and T_3_, a total of 10 DE/ESTs were found to be implied in the “Primary metabolic P” and one in the “Cellular P” (**Table S5**). Among the former group, *CHTS9987.b2* (β subunit of pyruvatekinase) and *CHTS11351.b1* (GAI-like protein), involved in processes such as canonical glycolysis and amino acid biosynthesis were upregulated at T_0_, while *EE258072.1* (phospho-glyceratekinase), active in the canonical glycolysis, was overexpressed at T_3_ (**Tables 6** and **7**). With regard to the latter group, *CHTS18557.b1* (UDP-galactose transporter 3), involved in the cell wall assembly, was overexpressed at T_0_.

#### The Temporal Expression Profiles of Expressed Sequence Tags/Genes Specifically Involved in the Carbohydrate Metabolism

Among the subset of 1,227 ESTs selected for being associated to the “Carbohydrate metabolic process” present in the chip (**Table 1**), 224 play a specific role in the metabolism of storage carbohydrates (**Table S6**).

**Figure 4:**
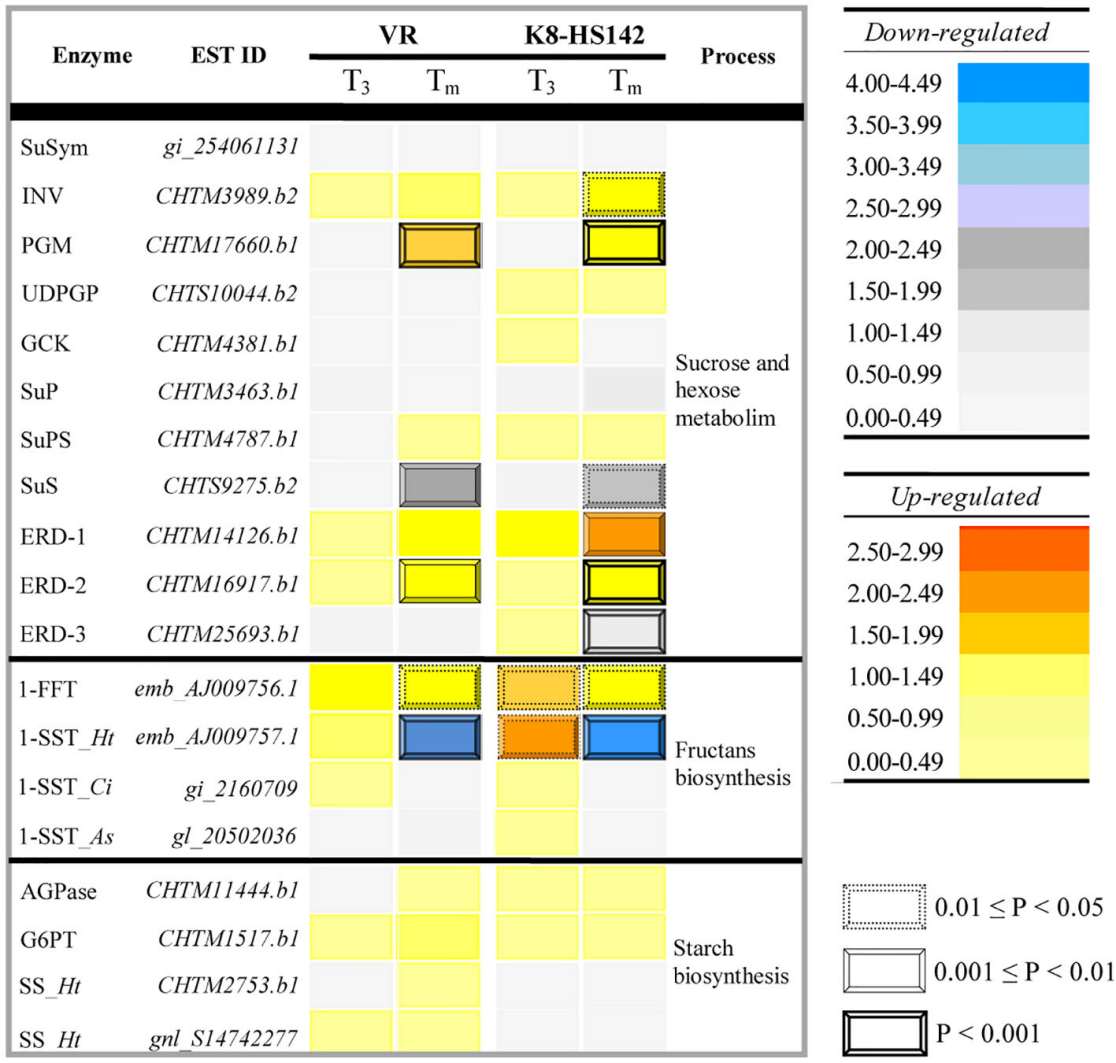
Temporal expression of the expressed sequence tags (ESTs) involved in the metabolism of storage carbohydrates in *Helianthus tuberosus* “VR” and “K8-HS142” clones. The relative emission levels of ESTs implied in the metabolism of sucrose, fructans, and starch at T_3_ or T_m_ compared to T_0_ reference stage (when tuberization begins), both in “VR” and “K8-HS142,” are displayed through a heat map. Magnitudes and statistical relevance of each EST contest are in the map. The statistical significance of the differences were tested according to the LIMMA. Key to genes. Simple sugars metabolism. *SuSym, Sucrose H^+^/symporter; SuP, Sucrose phosphatase; SuS, Sucrose synthase; INV, Neutral/alkaline invertase; GCK, Glucokinase; PGM, Phosphoglucomutase; UDPGP, UDP-glucose pyrosphorilase; ERD, Early Response to Dehydration 6 - H^+^/Glucose symporter*. Fructan polymerization. *1-SST, Sucrose:sucrose 1-fructosyltransferase; 1-FFT, Fructan:fructan 1-fructosyltransferase*. Starch metabolism and polymerization. *G6PT, Glucose 6-phosphate translocator; SS, Starch synthase. Ht, Helianthus tuberosus; St, Solanum tuberosum; Ci, Chicorium intybus; As, Allium sativum*.

Among them, 76 code for enzymes acting in the pathways of hexose metabolism, 79 in sugar transport through cell membranes, 56 in starch metabolism, and 14 in fructan biosynthesis. A total of 16 of these ESTs showed higher expression levels at one or more tuber stages in either “VR” or “K8-HS142” or both. The relative expression profiles of ESTs linked to specific enzymes operating in the hexose and sucrose metabolism, such as sucrose/H^+^ symporter (SuSym), alkaline/neutral invertase (INV), PGM, UDPG-pyrophosphorylase (UDPGP), glucokinase (GCK), sucrose phosphatase (SuP), sucrose phosphate-synthase (SuPS) and sucrose synthase (SuS), fructan biosynthesis, such as 1-SST and 1-FFT and starch metabolism, such as glucose 1-P adenylytransferase (AGPase), glucose-6P translocator (G6PT) and starch synthase (SS), are reported in the heat map in **Figure 4**, whereas in **Table S7** are given their absolute expression levels.

The ESTs for SuS (*CHTM9275.b2*), PGM (*CHTM8761.b1*), and INV (*CHTM3989.b2*) were DE between tuber stages (**Table 6**; **Figure 4**). The EST for SuS enzyme, operating in the breakdown of sucrose, was downregulated at T_m_ in comparison to T_0_ and T_3_ in both “VR” and “K8-HS142”, *PGM* peaked at T_m_ in both clones while *INV* only in “K8-HS142” (**Figure 4**; **Table 6**).

With regard to the genes for the biosynthesis of fructans, both the ESTs native from *H. tuberosus, 1-SST* (*Ht*_1-SST, *AJ009757.1*) and *1-FFT* (*AJ009756.1*), were DE between T_0_ and T_m_ in both clones (**Figure 4**; **Table 6**).

Probes for *1-SST*-EST from *Cichorium intybus* (*Ci*_1-SST, *gi_2169709*) and *Allium sativum* (*As*_1-SST, *gl_20502036*) also emitted signals (**Table S7**). These data suggest the presence of multiple but different gene members in *H. tuberosus* genome acting as sucrose:sucrose 1-fructosyltransferase. On this concern, we note that *Ci*_*1-SST* and *As*_*1-SST* ESTs showed dissimilarities at the nucleotide level (75.9% and 52.6%, of sequence similarity, respectively) to *Ht_*1-SST (**Table S8**).

The *1-FFT*-EST was more expressed at T_3_ and T_m_ in comparison to T_0_ in both “VR” (+143% and +205%) and “K8-HS142” (+181% and +174%), although with a different trend: in “VR,” it reached the maximum expression levels at T_m_, whereas in “K8-HS142,” it peaked at T_3_ (**Tables 6** and **S7**; **Figure 4**).

The EST*/*genes encoding for the enzymes operating in the starch biosynthesis, such as G6PT (*CHTM1517.b1*), AGPase (*CHTM11444.b1*), and SS (*CHTM2753.b1* and *S14742277.b1*), involved in glucose-6P mobilization from cytoplasm to amyloplast, turning glucose-1P into ADP-glucose, and glucose polymerization into starch, respectively, exhibited very low and no significantly different expession levels between either T_3_ or T_m_ and T_0_ (**Figure 4**; **Table S7**). At T_3_, the set of ESTs encoding for enzymes involved in the fructan polymerization (1-FFT, *Ht*_1-SST, *As*_1-SST, *Ci*_1-SST) were expressed to a higher average extent than those implied in the metabolism of hexose and sucrose (*SuSym*, *INV*, *PGM*, *UDPGP*, *GCK*, *SuP*, *SuPS*, *SuS*), which, in turn, were more expressed than the ESTs for starch biosynthesis (*G6PT, AGPase*, *SS*) (**Table S7**).

In order to verify the temporal expression profiles of the essential genes implied in the carbohydrate biosynthesis, the ESTs/genes encoding SuS and PGM enzymes acting in the sucrose breakdown and 1-SST and 1-FFT crucial for fructan polymerization were analyzed further by the q-RT-qPCR technique on independent RNA samples.

**Figure 5:**
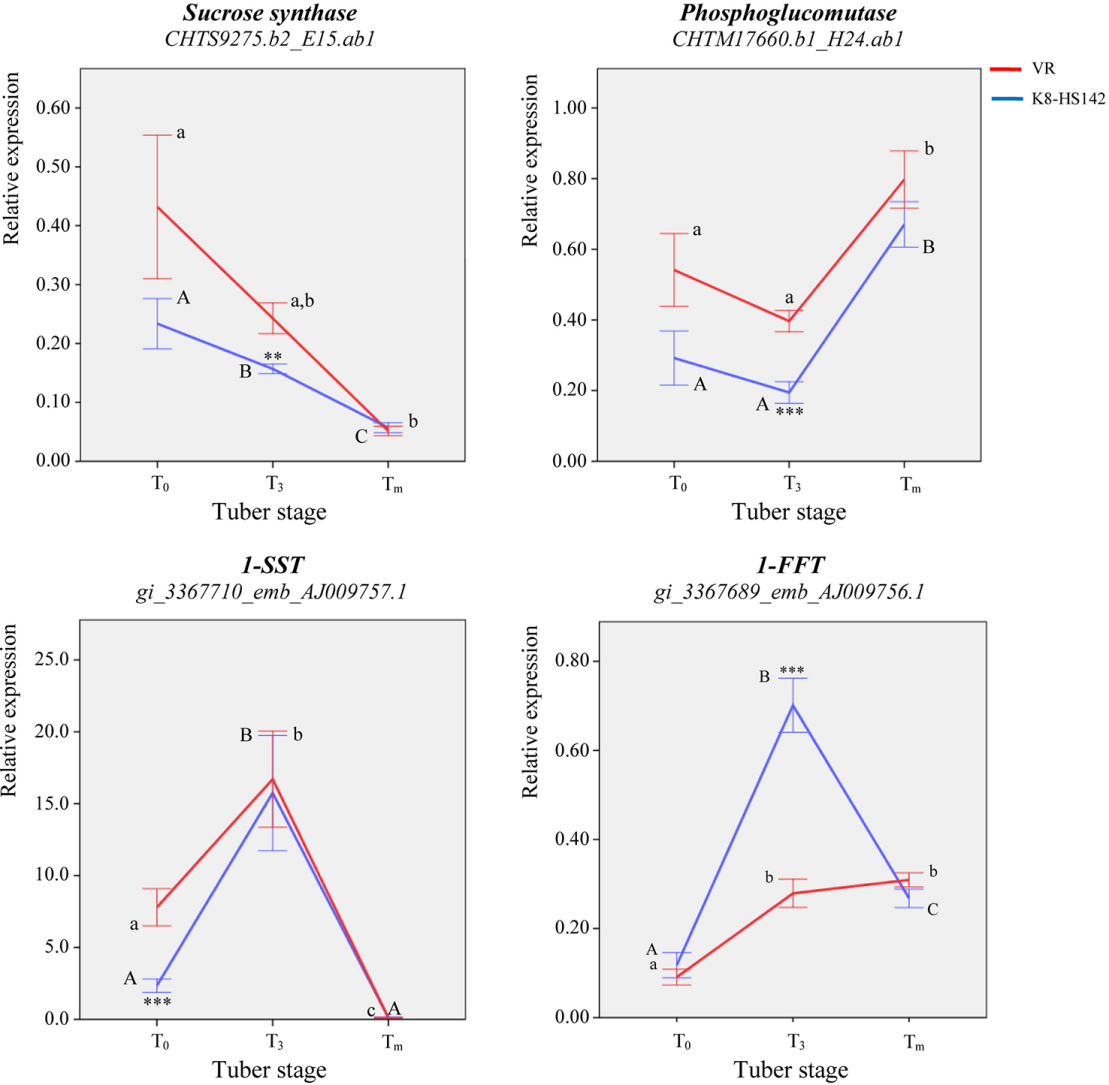
qRT- PCR analysis of key genes ruling the metabolism of storage carbohydrates in developing tubers of *Helianthus tuberosus* “VR” and “K8-HS142” clones. The temporal expression profiles by means of qRT- PCR at three specific tuber developmental stages (T_0_, T_3_, and T_m_) of genes implied in the cytoplasmatic metabolism of hexoses such as *SuS* and *PGM*, and fructan polymerization, *1-SST* and *1-FFT*, which were differentially expressed upon microarray analysis, are shown. The relative expression of each gene is calculated using the (2^-ΔCt^) algorithm. Statistically significant differences were assessed according to a two-way ANOVA between T_0_, T_3_, and T_m_ stages in “VR” and “K8-HS142” (indicated as *a*, *b*, *c* and A, B, C, respectively). Asterisks **, *** indicate the significance of the differences of transcript levels between clones at a given tuber stage (0.01 ≤ *p* < 0.01 and p < 0.001, respectively). The bars indicate ±1 standard deviation.

In keeping with that observed with microarray, the expression level of *SuS*-EST (*CHTS9275.b2*) diminished steadily during the entire tuber development in both clones (**Figure 5**). In line with microarray analysis, the expression of *PGM* (*CHTM8761.b1)* decreased slightly from T_0_ to T_3_ to significantly increase up to the final stage of maturation (**Figure 5**). Full concordance between data from qRT-PCR and microarray also emerged for the temporal expression profile of *1-SST*-EST (*AJ009757.1*) and *1-FFT*-EST (*AJ009756.1*) (**Figures 4** and **5**). Both analyses detected a higher expression level of the latter EST at T_3_ in “K8-HS142” and a quite linear increase during tuber development in “VR.”

Overall, the temporal expression profile of the two critical genes for the polymerization of inulin was similar in “K8-HS142,” where both peaked at T_3_, while differed in “VR” where *1-SST* peaked at T_3_ to significantly decrease at T_m_, whereas *1-FFT* steadily increased with tuber maturity.

The qRT-PCR analyses also confirmed that the relative expression of *1-SST*-EST was several order of magnitude higher than that of *1-FFT*-EST (**Figure 5**; **Table S7**).

## Discussion

The Jerusalem artichoke is an inulin-rich crop adapted to low-input agro-ecosystems (Cassels and Walsh, 1995; Mecella et al., 1996; Paolini and De Pace, 1997; Zhao et al., 2005; Kays and Nottingham, 2007; De Pace et al., 2010). The enzymatic basis of fructans metabolism and storage (Edelman and Popov, 1962; Dickerson and Edelman, 1966; Edelman and Jefford, 1968; Lüscher et al., 1996), the kinetics of 1-SST and 1-FFT enzymes (Koops and Jonker, 1994; Koops and Jonker, 1996), and genes coding for these enzymes are known for decades (Van der Meer et al., 1998).

A recent study has characterized the transcriptional levels of *1-SST* and *1-FFT* genes and measured the activity of the enzymes coded by these genes and by *fructan exohydrolase* (*FEH*s) genes, essential for inulin degradation, to analyze the sugar metabolism dynamics in tubers and bud eyes/shoots of a cultivar of *H. tuberosus* during germination (Jiao et al., 2018). The transcriptional profiles of the Jerusalem artichoke *FEH*s under stress was also investigated (Xu et al., 2015).

Here we analyzed, for the first time, the expression profile of key genetic determinants underlying tuber development, biosynthesis, and storage of complex carbohydrates in the tubers of two clones of *H. tuberosus* at three developmental stages.

The analysis of the transcriptome carried out here was performed by microarray because at the time the present study started, it was the most common choice of researchers to conduct transcriptional profiling experiments. Despite several superior benefits of RNA-seq over Microarray, RNA-seq technologies at that time were in fact more expensive, and data storage more challenging than microarray (Yan Guo et al., 2013; Zhao et al., 2014). We also note that because of the limited genetic resources available for *H. tuberosus*, the first assembly and annotation of its transcriptome by RNA-seq has been produced only very recently by Jung et al. (2014) who developed the first transcriptome dataset from five tissues, followed by Zhang et al. (2018) who assessed the transcriptomes in the tubers in response to salt stress.

Our study provides a model according to which the steady-state levels of *1-SST* and *1-FFT* transcripts are developmentally controlled and might represent a limiting factor for inulin accumulation. Target genes and tuber traits for breeding clones with enhanced levels of inulin have also been identified.

### Different Growth Rate and Pattern of Inulin Accumulation Between Jerusalem Artichoke Clones

The two selected clones of the Jerusalem artichoke show different growth habitus both in the epigeous and hypogeous organs. “VR” plants present multiple stems, those of “K8-HS142” are prevalently unbranched, “VR” forms pear-shaped tubers, those of “K8-HS142” are heavier and slender (**Figures 2B, C**). In “K8-HS142,” tuber differentiation starts later and tuber enlargement is slower than those in “VR” (**Figure 2A**). Conversely, the ripening phase is similar in length but dissimilar for what concerns the pattern of inulin accumulation. While at T_3_ the inulin content and its DP are significantly higher in “K8-HS142,” at T_m_, there are no differences between the clones (**Figures 3**). This because in “K8-HS142” inulin increases only slightly during the ripening stage.

Thus, the present study paves the way to agronomic trials to verify whether this pattern of inulin accumulation characterizes all clones with slender tubers and whether slender tubers produce more dry matter than pear-shaped ones at T_m_, regardless of farming systems adopted. Should these hypotheses be confirmed, then the target for the breeders would be to select genotypes with slender tubers on which the transcript levels of key genes such as *1-FFT* do not drop at T_m_ to maximize inulin yield (see below).

Finally, as far as starch content in tubers is concerned, only traces are found in mature tubers from both clones, confirming fructans as the dominant storage polysaccharides in *H. tuberosus*.

### Differentially Expressed Genes in the Rhizomes of the two Clones Rule Next Tuber Growth

A total of 60 and 11 DE/ESTs were detected between the two clones at the onset of tuberization and at the active tuber growth stage, respectively, whereas no DE/ESTs were found at the final stage of tuber ripening (**Table 2**). These data depict a picture where differences in tuber morphology and physiology between the clones are genetically determined since the early stages of rhizome enlargement.

In keeping with this observation, we note that the two clones differ for the expression of several genes related to cell development and growth, which at T_0_ are significantly more expressed in “K8-HS142” than in “VR” (**Tables 3**–**5**).

Much of plant physiology, growth, and development is controlled by the selective removal of short-lived regulatory proteins mediated by the 2-MDa protease complex (Smalle and Viestra, 2004) and we assessed that among DE/ESTs between clones at T_0_, there are those coding for ubiquitin-like modifier activating protein, shaggy related proteins, and non-ATPase subunit of 26S proteasome, all upregulated in “K8-HS142.” The E3 ubiquitin ligase belongs to the class of cytokinin downregulated genes (Rashotte et al., 2003). Because cytokinins play a fundamental role in promoting the tuber initiation (Palmer and Smith, 1969), the different extent in transcripts for an E3 ubiquitin ligase (*CHTS19020.b1)* at the initial stage of tuberization between the two clones could contribute to trigger the different precocity in tuber development. Yet, the differential expression of an auxin response factor (*CHTS10787.b1)* between the clones at T_0_ points toward the same conclusion.

Also, ESTs/genes controlling protein processes, such as translation, amino acid biosynthesis, protein stabilization, and modification, are upregulated in “K8-HS142” at T_0_, while those for protein depolymerization (i.e., aspartic protease and proteosome subunit β-type 5A) are downregulated in the same clone at initial tuberization (**Table 5**). This evidence led us to argue that processes related to protein biosynthesis take place to a different extent between the pear- and slender-shaped clones (**Figures 2B, C**; **Figures S1-A2, B2**). At T_0_, the two clones also differ for the expression of genes related to glycolysis and electron transport chain (**Table 3**), upregulated in “K8-HS142,” to suggest that a different rate of glycolysis might occur at the beginning of tuber development in “VR” and “K8-HS142” that, in turn, might affect the differences in the morphological and physiological traits of the tubers.

### Key Genes Related to the Development of Tubers in the Jerusalem Artichoke

By comparing the expression patterns between different Jerusalem artichoke tissues/organs by RNA-seq, Jung et al. (2014) pointed that the lowest number of DE genes (949) were noted between tubers at initial vs. maturation stage and concluded that “metabolic processes are similar at both stages of tuber development”.

In the present study, we aimed at detecting the genes liable for the processes of development and maturation of the Jerusalem artichoke tubers, regardless of the clone. To this end, we considered the DE/ESTs across the three developmental stages. From these comparisons, it emerges that the higher number of DE/ESTs is between the two extreme stages, T_0_ and T_m_, and these belong to the GO categories of “Cellular process,” including biological events dealing with cell growth, cell wall assembly and modification, water and hormone transport, protein modification, and carbohydrate metabolic processes (**Tables 6**–**8**).

In essence, our transcriptomic data fit well with the developmental processes of tubers, which in their initial stage need actively dividing and enlarging cells. Therefore, it makes sense the activation at T_0_ of genes playing an essential role in the cell growth, cell wall assembly, modification, and organization, water influx into cell and vacuole, and protein modification processes.

The finding that most of the DE/ESTs implied in cell growth (i.e., *CHTM25460.b1* and *CHTM2635.b1* for MAP3K ϵ protein kinase), cell wall assembly (CHTS18557.b1 and CHTM21118.b1 for UDP-galactose transporter 3), cell wall modification (*CHTS11585.b1* for pectin methylesterase), and water intake into the vacuole (*CHTS15962.b1* for tonoplast intrinsic protein) are concomitantly upregulated in both clones at this stage (**Table 7**) suggests them as the major players of these processes in both pear- and spindle-shaped clones. The overexpression at the initial tuberization of one EST for an auxin influx carrier protein is in keeping with what is supposed to occur and the role played by this hormone when tuber begins enlarging. In tobacco cultured cells, three-quarters of auxins enter the cells through the auxin influx carrier (Parry et al., 2001) and auxin in potato is vital for cellular water intake, differentiation and growth of tuber cells (Carlier and Buffel, 1955; Roumeliotis et al., 2012). It is also noteworthy that a member of the Zinc finger CCCH family, known to be a key regulator of plant development and to act at transcriptional and toposttranscriptional level (Wang et al., 2008), is significantly upregulated at T_0_ in both clones.

Conversely, ESTs encoding for a membrane steroid binding protein, a cryptochrome 1, and a polyubiquitin 2 protein are upregulated at T_m_ (**Table 7**). The membrane steroid binding protein has been characterized as a negative regulator of cell elongation in different plant species such as *A. thaliana* (Yang et al., 2005), cryptochrome 1 is an inhibitor of hypocotyls and plant growth in *A. thaliana* (Lin et al., 1996), and polyubiquitin is associated with apoptosis (Zhong et al., 2005), all of which are events expected to occur at the final stage of maturation. The concomitant overexpression at T_m_ of one EST for a histone deacetylase (*CHTS12733.b1)*, operating in the chromatin and gene silencing in both “VR” and “K8-HS142,” also heads to the limitation of cell growth at the final stage of tuber development.

However, ESTs encoding for the initiation factors 1, 4, and 5A (*CHTM6612.b1*, *CHTM1887.b1*, *CHTS15763.b1)*, involved in cell morphology (Mandel et al., 1995; Thompson et al., 2004; Gross and Kinzy, 2005) are overexpressed at T_m_ in both clones. This evidence suggests that the translational processes are fundamental until tuber maturation, regardless of the clone analyzed.

At this stage, among the more expressed ESTs implied in the carbohydrate metabolism there is one coding for an alkaline/neutral invertase and one for an aldolase (**Table 6**). The alkaline/neutral invertase catalyzes the sucrose breakdown into fructose and glucose. In carrots, the presence of this transcript occurs in all organs with slightly higher levels in developing organs, suggesting a widespread role of this enzyme in growth-related function (Sturm, 1999). The upregulation of an aldolase also fits with what it is expected to occur at the final stage of tuber development, since this enzyme negatively affects plant growth (Haake et al., 1998).

### A Model to Explain the Dynamics of Carbohydrate Storage in the Jerusalem Artichoke Tubers

The observed patterns of EST expression during tuber development in *H. tuberosus* provide new insights on the dynamics of storage carbohydrate accumulation in these auxotrophic organs.

**Figure 6:**
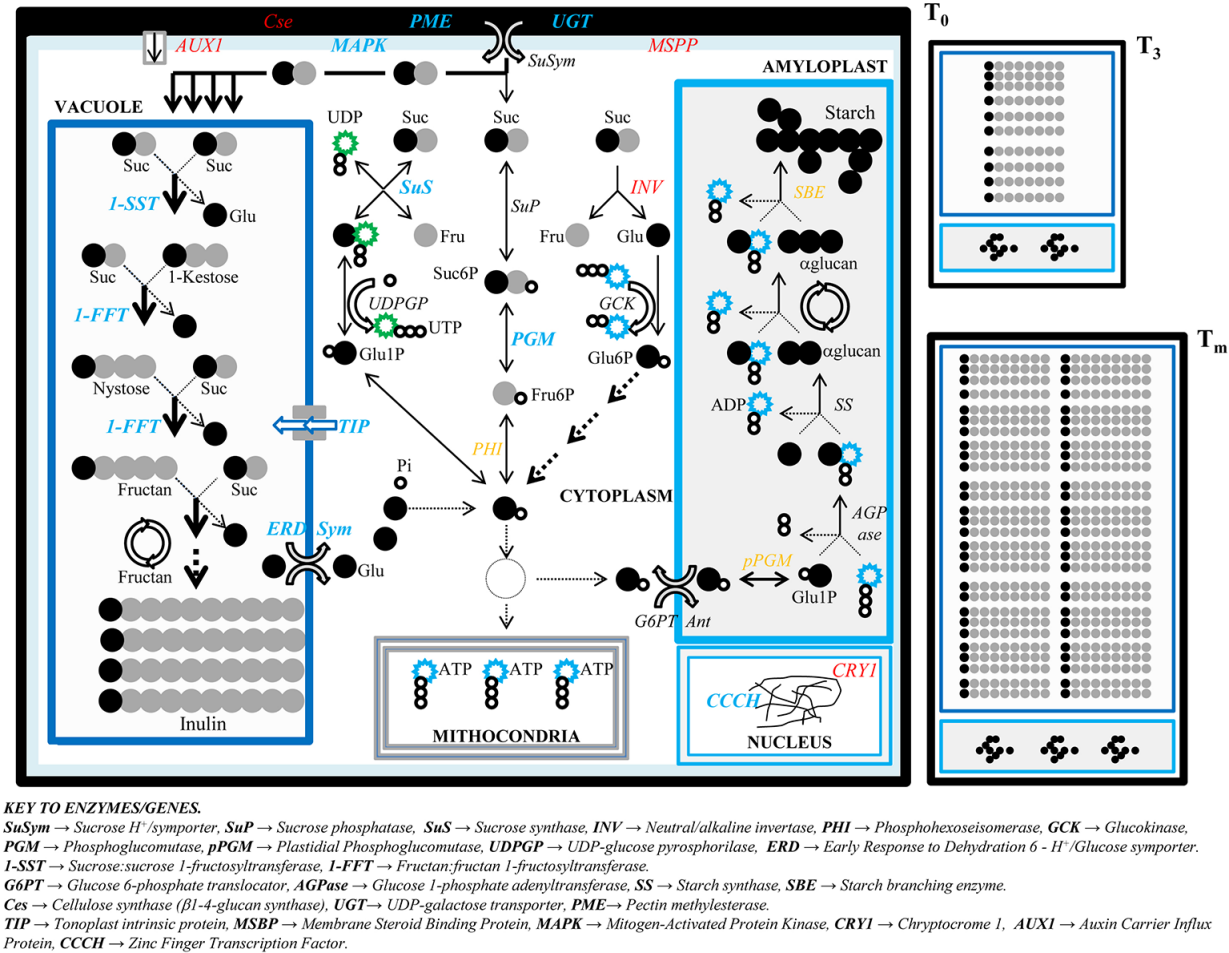
Model of inulin accumulation and cell growth in developing tubers of *Helianthus tuberosus*. A scheme is proposed to display the metabolic pathways of sucrose breakdown in the cytoplasm, fructan polymerization in the vacuole, and starch biosynthesis in the amyloplast from when rhizome starts enlarging (T_0_) until the stages of tuberous maximum growth rate (T_3_) and final tuber maturation (T_m_). While cell at T_0_, regarded as the reference stage in tuber development process, is displayed in the foreground, cells at the two next stages, when inulin deposition takes place moderately (T_3_) and massively (T_m_), are depicted smaller near the T_0_ cell. The main enzymes affecting the metabolism of carbohydrates as well as cell wall assembling and the overall cell growth are shown and placed in the cell compartments of their action. Emphasized arrows point to massive metabolic pathways. Enzymes whose genes are differentially expressed (DE) between T_0_ and T_m_ are reported in bold blue (if DE in both clones) or in red (if DE in one clone). The enzymes indicated in orange have genes not investigated in this study.

The tuber growth and carbohydrate production depend on sucrose phloematic transport from the leaves and stalk to the rhizomes (Edelman and Popov, 1962; Dickerson and Edelman, 1966). A strong initial sucrose supply is necessary to power a large fructose availability for fructan polymerization, but also to power and sustain the whole tuber growth. The expression level displayed by the EST encoding for the SuSym protein (*gi_254061131*), fundamental to enter the sucrose into tuber cells from phloem (**Figure 6**), fits with this model, since it is expressed all over the tuber developmental stages, although its expression is slightly higher at the initial phase of tuberization (**Figure 4**; **Table S7**).

The combination of 1-SST and 1-FFT enzymes purified from *H. tuberosus* can synthesize *in vitro* long-chain inulins from sucrose according to the classic two-step model (Edelman and Jefford, 1968; Lüscher et al., 1996; Koops and Jonker, 1996). The rate of 1-kestose (GF_2_) production by 1-SST is a function of the sucrose content in the tuber cells, and the enzyme deploys the upmost activity when the substrate concentration is elevated (around 1 M), as demonstrated by *in vitro* studies on long-term incubation time (80 h at 25°C). In addition to large amounts of GF_2_, other oligosaccharides are yielded, although at lower rate, as consequence of self-transfructosylations with GF_2_ and 1,1-nystose (GF_3_), such as GF_3_ and 1,1,1-fructosylnystose (GF_4_), respectively, all of them employed by 1-FFT to produce inulin (Edelman and Jefford, 1968) (**Figure 6A**). However, while the 1-SST-mediated GF_2_ synthesis is favored by high sucrose concentration, the 1-FFT-mediated second phase of fructan synthesis from GF_2_ to GF_n-1_ is competitively inhibited by sucrose (Koops and Jonker, 1994).

The expression level of the *Ht*_*1-SST* EST (*AJ009757.1*) keeps up with the progress of fructan accumulation over tuber growth. In fact, both the inulin content (**Figure 3**) and the expression level of *1-SST* peak at T_3_, as per microarray and qRT-PCR analyses (**Table 6**; **Figure 5**). This clear evidence of the tight relationship between the *1-SST* gene expression pattern and inulin accumulation rate in *H. tuberosus* mirrors what has been shown to occur between 1-SST enzyme and other classes of fructans (i.e., inulin neoseries, levans, and mixed-type levans) in other species (Edelman and Jefford, 1968; Vijn et al., 1997; Vijn and Smeekens, 1999).

Microarray results also indicate that two further genes for 1-SST, in addition to the unique *1-SST* transcript from the *H. tuberosus* described so far (Van der Meer et al., 1998), are expressed during tuber development (**Figure 4**; **Table S5**). The low sequence similarities with *Ht_1-SST* (75.9% for *Ci*_1-SST and 52.6% for *As*_1-SST) (**Table S6**) let us to argue that they are contributed by different *H. tuberosus* ancestral genomes. *H. tuberosus* is an allopolyploid species (2n=6x=102) derived from the merging of two duplicated genomes provided by the tetraploid *Helianthus hirsutus* with a third differentiated genome from the diploid *Helianthus grosseseratus* (Pustovoit et al., 1976; Bock et al., 2014). Our findings would suggest that the three different *1-SST* genes reflect as many as genomes contributed to shaping the hexaploid *H. tuberosus* and that they might be useful markers to trace its origin.

Jung et al. (2014) reported the higher expression levels of both *1-SST* and *1-FFT* at the tuber maturation stage rather than at the tuberous initial stage in cultivar, “Purple Jerusalem,” but no data relative to the progress of inulin accumulation in this cultivar were provided. The different timing of tuber collection and cultivars employed make it challenging to draw any parallelism between present and Jung et al.'s studies.

However, we note that in the present study the levels of *1-SST* transcripts are lower at T_m_ than any previous stage investigated in both clones and that the EST encoding for 1-FFT (*AJ009756.1*) increases at T_3_ in both clones to drop at T_m_ in “K8-HS142,” although to levels higher than at T_0_ (**Figure 5**). Thus, present work provides evidence that *1-SST* and *1-FFT* genes are regulated differently to each other and throughout the development of tubers, being *1-SST* highly transcribed since the beginning of tuberization until the active growth stage and *1-FFT* during the next phases of tuber ripening, contributing to inulin accumulation and elongation (**Figure 3**). The qRT-PCR data also show that the transcript levels of *1-SST* (*AJ009757.1*) are far higher than those of *1-FFT*_EST (*AJ009756.1*) from T_0_ to T_3_ (**Figures 5C, D**).

Since 1-SST and 1-FFT enzymes work in *H. tuberosus* at comparable activities (Koops and Jonker, 1994), the larger population of *1-SST* transcripts than *1-FFT* ones assessed during these stages is consistent with the need of high levels of 1-SST enzyme to process large amounts of sucrose to supply inulin stretching by 1-FFT.

Based on the kinetics and substrate specificity assays, the two-step model predicts *de facto* a synchronized activity of 1-SST and 1-FFT enzymes in *H. tuberosus*. Also in chicory roots, inulin accumulation results from a combined, but temporarily shifted, activity of 1-SST and 1-FFT: 1-SST activity is prominent in the early vegetative stage and then declines, while 1-FFT remains more or less constant throughout the growing season (Van Laere and Van den Ende, 2002).

Our study extends previous findings in that it points gene transcription and/or RNA turnover as a key regulatory process controlling the dynamics of inulin accumulation in *H. tuberosus*.

That the activity of the 1-SST and 1-FFT enzymes strictly mirrors the transcriptional levels of the respective genes has been shown in barley leaves, where it was found that 1-SST transcript and enzymatic activity are both subject to a rapid turnover, while the grasses specific fructan-6-fructosyl transferase (6-SFT) transcript and enzymatic activity were found to be much more stable (Nagaraj et al., 2004). Additionally, in the same study, it was shown that in young leaves, sucrose and light appear to be critical factors for the induction of these genes. Barley *1-SST* seems to have a lower threshold for the activation by cytosolic sucrose than *6-SFT* gene and *1-SST* transcripts are rapidly downregulated in the dark likely because of specific sequences in the UTR known to influence transcript stability.

In tubers, light is not expected to play a fundamental role, rather sucrose and other environmental factors might control the transcriptional rate, RNA stability, and/or enzymatic activity of 1-SST and 1-FFT. As an example, *in vitro* studies have shown that the two *H. tuberosus* enzymes have different optimal working temperatures, ranging between 20°C–25°C and 25°C–35°C for 1-SST and 1-FFT, respectively (Koops and Jonker, 1994; Koops and Jonker, 1996). At the medium latitudes, the stages of tuber enlargement and complete dimensional development occurs in late spring–early summer, whereas final tuber maturation occurs in summer, under higher temperatures. Thus, it is conceivable that temperature might be one of the drivers that control RNA transcription and turnover and, consequently, the enzymatic activity of 1-SST and 1-FFT.

As far as differences between clones are concerned, we note that the expression profiles for *1-SST* are similar, but the steady-state levels of these transcripts are significantly higher in “VR” at T_0_ (**Figure 5**). The expression profiles of *1-FFT* are conversely dissimilar (**Figure 5**). In “VR,” *1-FFT* markedly increases from T_0_ to T_3_ and steadily, albeit not significantly, from T_3_ to T_m_, whereas in “K8-HS142,” it peaks at T_3_ and drops at T_m_ at the same levels shown in “VR.” It is worth noting that at T_3_, both *1-FFT* and inulin levels are significantly higher in “K8-HS142” than those in “VR.” The data suggest a clone-specific regulation for *1-FFT* and open the avenue for targeted studies aimed at identifying polymorphisms in the regulatory regions of this gene between *H. tuberosus* clones. On this concern, we are tempted to speculate that *1-FFT* gene from “K8-HS142” is more prone than its orthologous in “VR” to a sucrose-mediated inhibition. Whatever are the reasons for *1-FFT* dropping at T_m_ in “K8-HS142,” our results are promising for breeding purposes since it appears conceivable to select and cross clones with the aim to maximize and sustain, up to the final stage of tuber development, the levels of *1-FFT*.

Finally, according to our model, the rate of sucrose supplying chain elongation decreases with tuber ripening. Thus, at T_m_, sucrose is mainly used to power other cellular processes such as glycolysis. Support to our hypothesis comes from the evidence that, in both clones, two out of three total ESTs for the ERD 6-like 6, peak at T_m_ (*CHTM14126.b1*, *CHTM16917.b1*) (**Table 6**; **Figure 4**). The ERD 6-like are H^+^ proton/symporter exporting glucose from vacuole to cytoplasm (Poschet et al., 2011; Hu et al., 2019). In turn, a high flow of sucrose to the cytoplasm might be responsible for the decreased levels of *1-FFT* in “K8-HS142.”

Starch is the principal storage polysaccharide in tuberous species grown worldwide such as potato, where a set of genes encoding for enzymes functioning in the cytoplasm, such as glucokinase (GCK) and glucose 6P/translocator (G6PT), or into the amyloplast, such as AGPase and starch synthase (SS), are switched on during the tuber development (Geigenberger, 2011) (**Figure 6**). Even though the sucrose imported from phloem into the tuber cells could potentially support the entire starch biosynthetic path in *H. tuberosus* (**Figure 6B**), here we show that starch is stored negligibly in its mature tubers. In line with this, we note that the ESTs for starch biosynthesis show emittance levels lower than those for fructans (**Table S7**), and their expression levels are not significantly different across tuber stages (**Figure 4**).

The expression profiles of ESTs encoding for SuS (*CHTS9275.b2*) and PGM (*CHTM8761.b1*) enzymes could also aid to address the issue of low storage of starch (**Figures 4** and **5**). In potato tubers, SuS is the major responsible for sucrose breakdown (Zrenner et al., 1995), and the observation that *SuS*-EST transcripts are accumulated massively at T_0_ in both *H. tuberosus* clones let us to argue that at the initial stage of tuberization, the glucose released by this enzymatic activity will propel glycolysis for generating ATP and power cell growth (**Figure 6**). Notably, of the two corn paralogous genes that encode isoenzymes of SuS, only one is needed for generating precursors for starch biosynthesis, whereas the other provides the substrate for cellulose biosynthesis (Chourey et al., 1998).

On the contrary, the massive expression of PGM at T_m_ would address the remaining glucose, not more necessary to power tuber development processes *via* glycolysis, toward the formation of sucrose and inulin, guaranteeing the minimum loss of unexploited glucose. At this regard, we note that in potato tubers, the expression of the *St*cPGM gene in antisense orientation led to the accumulation of sucrose in the amyloplast and the reduction of starch (Fernie et al., 2000).

**Figure 6** provides a model to describe the parallel progresses of inulin accumulation and cell organization throughout the development of *H. tuberosus* tubers in which are highlighted the DE/ESTs related to carbohydrate metabolism and cell growth and modification between the stages of tuber formation and maturation.

## Conclusions

New insights on gene expression and metabolism of storage carbohydrate accumulation in tubers of *H. tuberosus* stems from the present study, namely:

- The temporal expression of *1-SST* and *1-FFT* genes and the dynamics of inulin accumulation are synchronously regulated according to the growth stages.
- The presence of three divergent *1-SST* genes reflects the allopolyploid origin of the hexaploid *H. tuberosus*.
- The different expression pattern throughout tuber development of *1-FFT* between “VR” and “K8-HS142” clones calls for a different regulatory mechanism of inulin accumulation between genotypes differing in tuber morphology and physiology;.
- The negligible expression of genes for the starch biosynthesis explains the undetectable starch content in tubers.
- The dynamics of tuber development is accompanied by the sequential overexpression of genes related to cell growth, proliferation, enlarging, and elongation at the initial phase of differentiation followed by that of negative regulators of these processes at maturation.
- The extent to which the expression of genes such as those related to cell growth and protein processing occurs at the initial phase of tuber differentiation is credited to mark the difference between pear- and slender-shaped clones. In turn, this difference might result from a different biosynthetic and/or exploitation rate of glucose between clones.

Overall, the comparison of transcriptomic and inulin profiles between tubers of different shapes throughout their developmental stages allowed us to depict that a small set of genes command relevant differences in timing and extent to which tubers develop and accumulate carbohydrates. These findings set the stage for a more in-depth analysis focused on regulatory genes controlling the different patterns of growth between clones.

Present findings provided a framework not only to understand the molecular mechanisms of tuber development and fructan biosynthesis in *H. tuberosus* but also to breed inulin-rich clones under low-input farming systems.

## Data Availability Statement

The data reported in this study have been deposited in NCBI's Gene Expression Omnibus and are accessible through GEO Series accession number GSE132955 (https://www.ncbi.nlm.nih.gov/geo/query/acc.cgi?acc=GSE132955).

## Author Contributions

MB designed the experiments and collected and analyzed the data from the experimental plots. MB and DV isolated and prepared RNA for microarray and qRT-PCR analyses. AF, PT, EZ, and MB under the supervision of MD performed microarray analyses. FP and FD performed qRT-PCR analyses. MB and FP wrote the manuscript with input from the other coauthors.

## Funding

This work was financed by the Italian Ministry of Agriculture (MiPAAF) as part of the *ENERBIOTOP* Project aimed to study “The biomass produce from the Jerusalem Artichoke (*Helianthus tuberosus* L.) to convert in a sustainable manner into biofuels replacing analog ones from hydrocarbons.”

## Conflict of Interest

The authors declare that the research was conducted in the absence of any commercial or financial relationships that could be construed as a potential conflict of interest.

## Footnotes

1 http://cgpdb.ucdavis.edu/database/DatabaseDescription.html

2 http://www.mged.org.miame

3 http://ddlab.sci.univr.it
