# Supplementary material for: Whole-Transcriptome Analysis Unveils the Synchronized Activities of Genes for Fructans in Developing Tubers of the Jerusalem Artichoke
Source: Front Plant Sci. 2020 Feb 21;11:101. doi: 10.3389/fpls.2020.00101 (PMC7046554; doi:10.3389/fpls.2020.00101)
Supplement: Supplementary file 8 [file Table_8.docx]

**Table S8. Sequence identity of *1-SST* gene*s*.**

| EST/gene | *1-SST Ht*  *embAJ009757.1* | *1-SST Ci*  *gb81520.1* | *1-SST As*  *gbAY098442.1* |
| --- | --- | --- | --- |
| *1-SST Ht*  *embAJ009757.1* |  | 0.759 | 0.526 |
| *1-SST Ci*  *gb81520.1* |  |  | 0.514 |

The table reports the sequence identity (%) calculated by the BioEdit Sequence Alignment Editor between the EST*s* for 1-SST enzyme from *H. tuberosus* (*Ht*), *C. intybus* (*Ci*) and *A. sativum* (*As*) on which oligos were designed and spotted onto the GeneChip.
