# Supplementary figures and images for: Whole-Transcriptome Analysis Unveils the Synchronized Activities of Genes for Fructans in Developing Tubers of the Jerusalem Artichoke
Source: Front Plant Sci. 2020 Feb 21;11:101. doi: 10.3389/fpls.2020.00101 (PMC7046554; doi:10.3389/fpls.2020.00101)

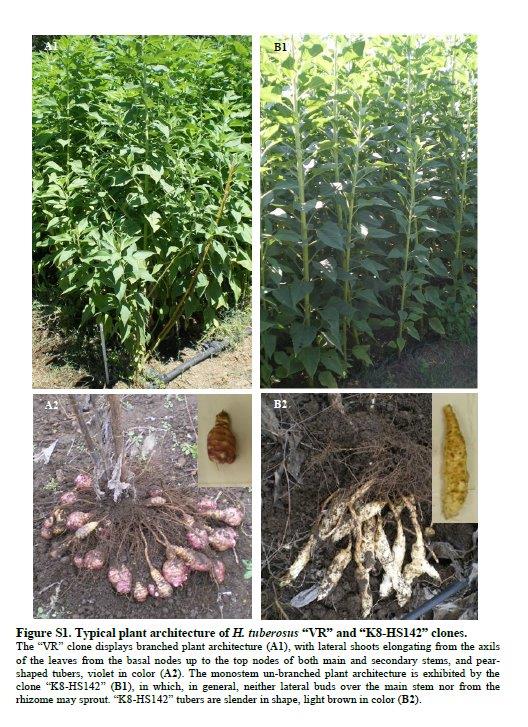

Supplement: Supplementary file 9 [file Image_1.jpeg]

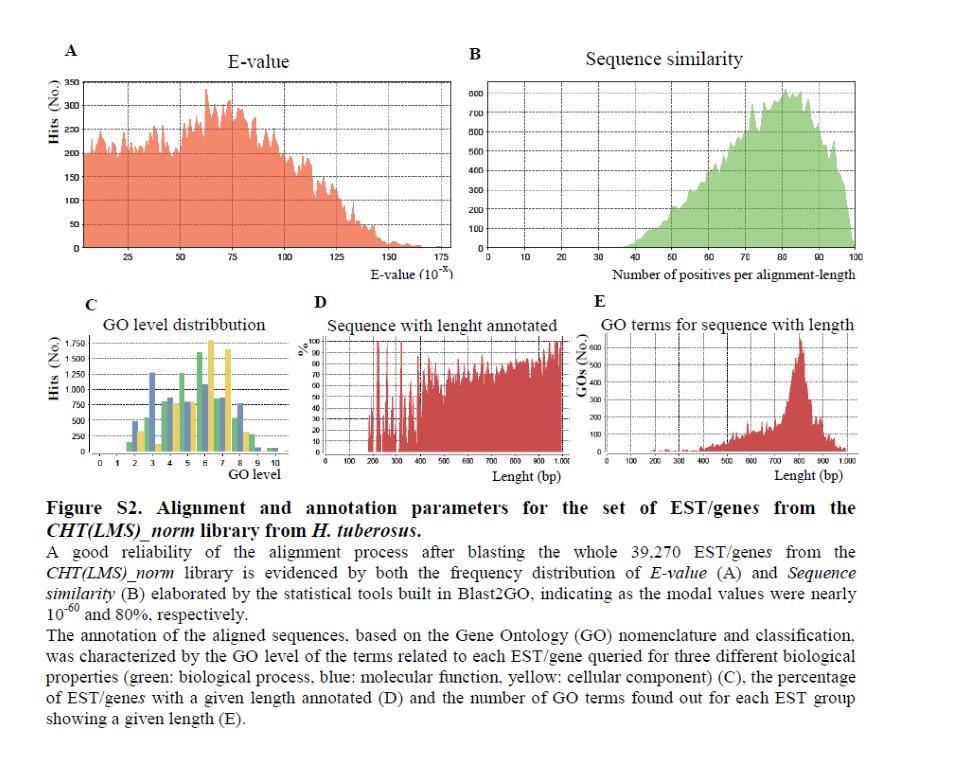

Supplement: Supplementary file 10 [file Image_2.jpeg]
